# Carbon Footprint Reduction by Transitioning to a Diet Consistent with the Danish Climate-Friendly Dietary Guidelines: A Comparison of Different Carbon Footprint Databases

**DOI:** 10.3390/foods11081119

**Published:** 2022-04-13

**Authors:** Ellen Trolle, Matilda Nordman, Anne Dahl Lassen, Tracey A. Colley, Lisbeth Mogensen

**Affiliations:** 1 Nutrition, Sustainability and Health Promotion Group, National Food Institute, Technical University of Denmark, Kemitorvet, DK-2800 Kgs Lyngby, Denmark; matnor@food.dtu.dk (M.N.); adla@food.dtu.dk (A.D.L.); 2 Quantitative Sustainability Assessment (QSA) Group, Sustainability Division, Department of Technology, Management and Economics, Technical University of Denmark, DK-2800 Kgs Lyngby, Denmark; trco@dtu.dk; 3 Department of Agroecology, Faculty of Technical Sciences, Aarhus University, Blichers Allé 20, DK-8830 Tjele, Denmark; lisbeth.mogensen@agro.au.dk

**Keywords:** carbon footprint, greenhouse gas emissions, food based dietary guidelines, plant-rich diet, climate impact, sustainability

## Abstract

Dietary transitions are important for combating many of the environmental challenges humanity is facing today and reducing the global burden of disease. Different dietary patterns are associated with substantially different carbon footprints (CFs). This study aims to estimate the potential CF reduction on a transition from the current Danish diet to a plant-rich diet consistent with the Danish food-based dietary guidelines (FBDG) and to compare results obtained from the use of two different CF databases. Dietary intake data for adults aged 18–64 years from the national dietary survey 2011–2013 were used to calculate the CF of the current diet, and this was compared with the estimated CF of the plant-rich diet modelled for the FBDG. Calculations were carried out using an attributional life cycle assessment (LCA) database (AU-DTU data) and compared to calculations using a top-down hybrid consequential LCA database (BCD data). The transition from the current diet to the plant-rich diet showed a substantial estimated CF reduction of 31% with AU-DTU data, and a greater reduction with BCD data (43%). Ruminant meat reduction was the largest contributor to this CF reduction, especially with the use of BCD data, and other animal-based foods also contribute considerably to the CF reduction, especially with AU-DTU data. These results indicate that the choice of LCA methodology and CF database is important in estimation of dietary CF and for the development of guidelines to promote dietary change.

## 1. Introduction

The current food system has been described as unsustainable as it contributes to increased environmental burdens which approach or exceed planetary boundaries, potentially leading to irreversible and harmful changes in earth systems [1,2]. In particular, analyses have focused on the negative impacts of greenhouse gas (GHG) emissions, but other critical factors are affected, such as land use, fresh water scarcity, nitrogen and phosphorous use, and biodiversity impact [3].

The food system accounts for approximately 21% to 37% of overall global anthropogenic GHG emissions and it is estimated that, without interventions, a further increase of 30–40% is likely by 2050 [4,5]. Instead, a considerable reduction in GHG emissions is needed. The Glasgow Climate Pact (COP26) reaffirms the Paris Agreement goal of limiting the future increase in global temperature to 1.5 degrees and focuses on the commitments from participating countries [6].

To support this commitment, in 2018, the Danish parliament agreed upon the goal of a 70% reduction in GHG emissions by 2030 compared with 1990 levels and this was confirmed by law [7]. In line with this, “The Official Danish Dietary Guidelines–good for health and climate” were launched in January 2021 [8] to contribute to the needed reduction in GHG emissions through changes in food consumption while at the same time ensuring a healthy and nutritionally adequate diet. The scientific basis for the new FBDGs included the modelling of a more plant-based diet, i.e., “the Danish Adapted Healthy Plant-Rich Diet” [9]—for simplicity, referred to as “the plant-rich diet” here. The plant-rich diet was developed to take into account the evidence on the relationship between food intake and disease risks (ref) and adequacy of nutrients, in accordance with the Nordic Nutrition Recommendations 2012 [10]. The plant-rich diet has a low amount of total meat (especially beef) and discretionary foods; a relatively high amount of vegetables, legumes, fruits, nuts, seeds, wholegrain products and potatoes; and moderate amounts of fish, milk and dairy products, eggs and vegetable oils [9]. All the food groups (except for discretionary food) have a role in contributing to health and nutrient intake.

The effect of changes in current Western dietary patterns towards more sustainable patterns based on GHG emissions—calculated as the carbon footprint (CF)—has been estimated in several studies. For example, exchanging ruminant meat for meat with a lower GHG impact or partial replacement of meat and dairy with plant-based products has been estimated to reduce the total diet CF by 20% to 40% [11,12]. Likewise, a reduction potential of 25% in GHG emissions for food purchased by both child care centers [13] and nursing homes [14] has been identified, through similar changes to those suggested by the official FBDG, e.g., reducing the amount of meat, especially, ruminant meat, and increasing the amount of legumes in meals.

The calculated CF from different dietary patterns and dietary changes depends both on the relative content of food groups in the diet, and on specific foods within food groups, as the CF of food items can vary between foods from different food groups and vary significantly between foods within the same food group. Furthermore, the CF data used for the calculation also have the potential to affect the results. The CF data can vary depending on the methodology and the data used for the calculation of the CF. CF of foods vary depending on their specific production systems. In addition, CF vary depending on methodological choices, such as the system boundaries used, i.e., which elements in the food product’s life cycle are included from farm or retailer gate. Another methodological issue is whether the studies include the GHG emissions from land use change (LUC) and whether LUC are estimated as direct (dLUC) or as indirect land use change (iLUC), which will affect the CF of food items [15].

Different approaches have been used to establish the CF of foods, where attributional and consequential LCA (aLCA and cLCA) are two fundamentally different approaches [16]. Typically, aLCA will use average data, whereas cLCA will used marginal data. ALCA estimates the impact of the actual production, whereas cLCA estimates the consequences of producing an extra unit [16]. An aLCA will typically be based on a bottom-up approach, where studies on specific food items take all processes within the defined system boundaries into account. This is the approach used in the European Commission Product Environmental Footprint Category Rules [17]. Conversely, the top-down approach uses input–output analysis, which is an economic method, and assumes a direct correlation between cost and environmental impact. This approach is based on statistical data, e.g., at the national level, which are then divided to represent the specific processes of the product in question [18]. A report comparing top-down and bottom-up approaches and different scales (e.g., consumption and consumers in the EU) showed convergence of the results, although also concluding that generally top-down approaches are overestimating impacts [19]. Sugimoto et al. showed that the diet-related GHG emissions of Japanese diets differed according to three calculation methods: a literature review of life cycle assessment studies of foods, and production- and consumption-based input–output tables (IOT) using the Japanese IOT [20]. Others have demonstrated variation in the CF of foods [21] and in the estimated CF of diets [22]. However, literature describing the impact of the choice of CF data is sparse and the selected approach might be important in relation to decisions regarding a future sustainable food system.

The main objective of this study was to estimate the CF reduction on a transition from the current Danish diet to the plant-rich diet as recommended by the “Official dietary guidelines–good for health and climate”, including the contribution of different food groups to the total CF of the diets. An additional objective was to provide updated CF data of foods on the Danish market based on a traditional attributional LCA (bottom-up) approach and to compare results using this method with results when using CF data on foods based on a consequential LCA, top-down hybrid approach.

## 2. Materials and Methods

The CF was estimated for two different diets: (1) the current diet for Danish adults 18–64 years and (2) the modelled plant-rich diet, which is the reference diet behind the Danish FBDG. Two different databases including CF data of each food item were used to estimate the CF of these diets: (1) a database developed by Arhus University together with the Technical University of Denmark (AU-DTU data), which is based on a review of existing LCA studies using aLCA and a bottom-up approach (see Section 2.3) and (2) the Danish Big Climate Database (BCD data), developed by the climate think tank CONCITO in collaboration with 2.-0 LCA Consultants, which makes use of cLCA and a top-down hybrid approach using I-O data [18,23] (see Section 2). The CF of food is reported as kg CO_2_-equivivalents (CO_2_-eq) per kg food.

To enable comparability of the CF of the diets, the same system boundaries were used for the two data sets, i.e., CF data were aligned with food intake data, such that both sets of CF data represent the same functional units. This required that the CF of foods were adjusted to represent the edible part of the food, i.e., excluding bones, peels, etc. Furthermore, for some foods, the intake was given in raw weight and the CF values of the raw foods were used (e.g., most meat and fish) and the CF contribution from cooking was added. For some foods, the CF values of prepared foods, such as bread, sausages, cold cuts and smoked or canned fish products were used, taking weight loss or gain during food production and preparation into account.

The system boundaries in this study were chosen to represent (1) the retail gate, including primary production and the following processes: processing, transport, packaging, storage, losses throughout the chain until retail gate, and contribution from cooking at home and (2) the household level, i.e., estimates at the retail gate including the impact of waste at the household level and contribution from cooking at home.

### 2.1. Current Dietary Intake Data

The current food intake was based on results from the Danish National Survey on Dietary habits and physical Activity (DANSDA) 2011–2013, a nationwide, cross-sectional survey of diet and physical activity in a representative sample of individuals in the Danish population [24]. Intake of food and drink was collected from a representative sample of 3946 those aged 4–75 years. The present study included data for adults aged 18–64 years (N = 2492). The DANSDA 2011–2013 survey is described in more detail elsewhere [24]. In short, participants recorded their food intake for seven consecutive days using a pre-coded food diary, household measures (cups, plates, etc.) and a picture booklet with images of 4–6 portion sizes for selected foods. Recorded intakes were converted into ingredients, resulting in a list of 427 food items found in the Danish food composition database [25], representing both raw (e.g., apples) and processed foods (e.g., cold cuts and bread).

Food intake was estimated as intake per person per day. In addition, food intake was calculated per 10 MJ in order to remove variation due to energy intake. According to Nordic Nutrition Recommendations 2012, 10 MJ is the approximate daily reference energy requirement of an average adult with a moderate physical activity level across all ages and genders [10]. Food intake was aggregated into major food categories according to their environmental impact and nutritional significance in a plant-rich diet.

### 2.2. The Danish Adapted Plant-Rich Diet

The Danish adapted plant-rich diet [9] was chosen because it is the reference diet in the “The Official Danish Dietary Guidelines–good for health and climate” (FBDG) [8]. The plant-rich diet is an omnivorous diet that aims to limit—but not exclude—meat and other animal-based products. The plant-rich diet was designed for those aged 6–65 years (per 10 MJ) and to comply with the EAT-Lancet Commission’s global reference diet [2] but taking national food availability and culture into account. This was achieved by using Danish food composition data and food consumption data for 3189 adults aged 15–75 years [24,25] as the starting point for the modelling. These data include processed foods, discretionary foods and beverages in the diet according to current consumption patterns. In addition, the modelled intake was adjusted to incorporate the scientific evidence on the relationship between food intake and disease risk, including the scientific background of the former national FBDG [26] and scientific updates, and with the recommended nutritional quality of the diets for groups of individuals 6–65 years of age with a heterogeneous age and sex distribution in accordance with the Nordic Nutrition Recommendations [10]. Lassen et al. 2020 [9] describe the modelling of the plant-rich diet in detail, including advice on choosing more sustainable seafood, such as herring and mackerel, based on data from Hallström et al. 2019 [27]. The official FBDG includes advice on choosing more environmentally friendly fish and making use of official labels, such as the NaturSkånsom (can be translated to “Fishing with Care”), fish produced organically, the private Marine Stewardship Council (MSC) label for sustainably caught fish and the Aquaculture Stewardship Council (ASC) label for sustainably farmed fish.

### 2.3. The Carbon Footprint of Food from AU-DTU Database

The AU-DTU CF data are based on existing LCA data from literature regarding CF from farming and processing in combination with standard factors from other sources to obtain the CF at the desired system boundaries for each individual food item for which dietary intake data exist. LCA data were harmonized so that all CF estimates included the same life cycle stages to the extent that this was possible. Data include the CF from primary production and processing, packaging, transport (from production to retail), storage, and cooking at home to provide the total CF at the retailer gate and at the household. Food losses were taken into account using waste factors throughout the food chain. The CF from land use change was not accounted for as it was not included in most of the underlying CF studies. The compilation of CF data of food items for estimation of CF of different dietary patterns was carried out as a collaboration between researchers from Aarhus University (AU) and two departments at the Technical University of Denmark (DTU): National Food Institute (DTU Food) and Department of Technology, Management and Economics (DTU Man).

#### 2.3.1. Primary Production and Processing

Values for the CF from primary production (farming) and processing of foods were based on existing literature in the form of published LCA studies, reviews, and existing databases typically based on a bottom-up approach and aLCA (Supplementary Materials Table S1) [21,28,29,30,31,32,33,34,35,36,37,38,39,40,41,42,43,44,45,46,47,48,49,50,51,52,53,54,55,56,57,58,59,60,61,62,63,64,65,66,67,68,69,70,71,72,73,74,75,76,77,78,79,80,81,82,83,84,85,86,87,88,89,90,91,92,93,94,95,96,97,98,99,100,101,102,103,104,105,106,107,108,109,110,111,112,113,114,115,116,117,118,119,120,121,122,123,124,125]. The references that were prioritized for use were those that represent the production systems/countries that the foods that Danes typically consume come from. For Danish foods, we used LCA data on Danish food production systems, if available. For the CF of imported foods, we used data from the production systems estimated to correspond to products in the Danish market whenever possible. For example, we estimated that 80% of the tomatoes sold in the Danish market are grown in greenhouses and therefore we calculated the CF for tomatoes as an 80/20 combination of greenhouse grown and field grown, respectively. When several relevant references were found, the estimated value was based on all references (Supplementary Materials Table S1). In some cases, the value was based on one old reference but supported by newer references, such as eggs, where Moberg et al. [39] found similar values as referred in Supplementary Materials Table S1. When the CF from primary production of a product was not available, values were estimated from similar products, aiming at similarity in production system.

The CF from processing of each food was obtained from the same literature references as primary production, when appropriate (e.g., bread), or through addition of estimated standard values. For all dried legumes, nuts and seeds, an estimated standard processing value of 50 g CO_2_-eq was added (estimated from Landquist and Woodhouse [43]). For processed vegetables and fruit (i.e., frozen, canned and dried products), a standard value of 250 g CO_2_-eq was added to the primary production value, based on data from Landquist and Woodhouse [43].

#### 2.3.2. Packaging

Standard values for packaging were based on a review of available literature and overall estimates of the CF of packaging material per kg food [31,39,122,126,127,128,129,130,131,132,133]. Values were assigned based on general estimates of the types of packaging material used for different food items and the CF associated with production of the material (Supplementary Materials Table S2).

#### 2.3.3. Transportation

To estimate the CF of transportation for each food item, we distinguished between the CF contribution from local transportation including transport from primary production to processing and to supermarket and the extra contribution from import of foods to Denmark. As a rough estimate for the total CF from transport, we summed the CF from local transport and the CF from import (for the proportion of foods that is assumed to be imported). The proportion of Danish/imported food was based on Mogensen et al., 2020, Supplemental Table S8 [50]; and where a product constituting a smaller proportion of the diets was missing in that reference, we used a 50/50 proportion. Transportation by the end consumer from retailer to home was not included.

For local transport, we assigned products either a “high” or a “low” CF value. When the product is minimally processed and does not require refrigerated transport (e.g., fresh fruit and vegetables, nuts, seeds and legumes), we gave the product a low value for local transport, whereas the ‘high’ CF value for transport was used when the product requires more processing and refrigerated transport (e.g., meat). For dairy, the CF from local transport was based on the transportation of raw milk (milk equivalents) needed for producing different dairy products, as described by Flysjö et al. [31]. The “low” value, 55 g CO_2_/kg food, corresponds to 220 km by a 10–20-ton truck [50]. The “high” value was assumed to be twice the low value, 110 g CO_2_/kg.

For the CF from transport from import, we added 200 g CO_2_/kg food for the proportion of imported food as a rough estimate based on a mixture of countries of origin. The CF from transport was estimated to approximately 100 g CO_2_/kg for food from neighboring countries such as Germany, Sweden and the Netherlands, and up to approximately 300 g CO_2_/kg for food from overseas locations such as South America, Australia, and Africa [50]. Though for foods such as banana and coffee, we used the high value of 300 g CO_2_/kg food for import transportation.

In the future, the CF contribution from transport can be updated if data on countries of origin become available for each food item.

#### 2.3.4. Cooking

The CF from cooking at home was included in the calculations of the total CF. The proportion of the food that is cooked, the energy needed for cooking different types of foods, and the CF associated with energy use were used for the calculations.

The energy use required for cooking the different food items was based on previous work by Mogensen et al. 2020 [50]. The CF associated with the use of electricity for energy was updated to reflect present-day electricity supply, which in Denmark relies more than previously on the use of renewable energy and thereby the CF is estimated to be 343 g CO_2_/kwh [134]. For different foods, the CF associated with cooking 1 kg raw food is presented in Supplementary Materials Table S3. The estimated proportion of each food that is cooked before eating was based on expert knowledge and on data from DANSDA 2011–2013 [24]. No cooking was applied to processed foods, e.g., bread, dairy, oils and processed meat, fish, vegetable and fruit products.

#### 2.3.5. Storage

The CF for the storage of foods in retail shops and homes was estimated based on the type of storage applied to different foods and the GHG contribution from different types of storage. GHG contributions were based on previous work by Mogensen et al., 2020 [50] and updated with figures for present-day Danish energy supply. The assumed type of storage as well as the CF applied to different types of foods are presented in Supplementary Materials Table S4.

#### 2.3.6. Food Losses

The total CF of a food item is affected by losses throughout the product’s life cycle, both unavoidable losses in the form of inedible parts, e.g., peels and bones, and avoidable food waste in the chain, i.e., in production, retail and households. The CF of 1 kg edible food is higher, since more food needs to be produced to account for both avoidable and unavoidable losses.

Waste fractions for the avoidable losses applied to different foods were based on values presented in Mogensen et al., 2020 Supplementary Table S6 [50].

When accounting for unavoidable losses, it is important to distinguish whether literature values for the CF of foods represent only the edible part of the food or whether indelible parts (e.g., peels and bones) are included. Unavoidable losses in the form of inedible parts typically need to be deducted from raw fruit and vegetables and meat, while for other foods unavoidable losses are included in the CF from processing. Factors for unavoidable losses for fruit and vegetables were taken from the Danish food composition database [25], except potatoes, for which a factor of 15% was estimated. Unavoidable losses were estimated to 10% for eggs [50], 23% for chicken [73] and 1% for beef, since the majority of beef is consumed as minced meat, which has an unavoidable loss of 0%. All other foods, including pork, fish, cereals, nuts, seeds, legumes, dairy, fats, beverages, discretionary products, and processed foods had an unavoidable loss of 0% because inedible parts are assumed to be removed at the processing stage.

### 2.4. Carbon Footprints of Food from the Big Climate Database (BCD)

The BCD contains CF values of 500 food items as they appear at the retailer on the Danish market [23]. The methods used to produce the data in the BCD are described by CONCITO in a methodology report [18]. Data include the CF from agriculture, food processing, packaging, transport, retail and iLUC, and the total CF at the retail gate. Since each life cycle stage is reported independently and separation of life cycle stages is possible, we retrieved CF data at the retail gate both excluding and including iLUC. To align life cycle stages and functional units with dietary intake data and AU-DTU CF data, we added unavoidable loss fractions and the CF from cooking at home where relevant, using the same values as described in Section 2.3.

To calculate the climate impact of the diets using data from the BCD, we linked each of the 427 food items in the dietary intake data with food items in the BCD. This was achieved following a stepwise process. First, food items were automatically linked by direct match by name, i.e., where the names in the dietary intake data and the BCD matched 100%. Second, we proceeded by manual data enrichment. Products in the dietary intake data were compared with products in the BCD and where they were deemed to have convincing similarity (i.e., essentially the same product), a manual match was made. After stages 1 and 2 of the linkage process, 73% of the food products were linked. Third, remaining food items were linked by identifying the closest corresponding product or average of a category of products in the BCD, aiming at similarity in production systems.

In the BCD, the CF values for beef and pork vary substantially depending on the cut of the meat: 31–152 kg CO_2_-eq/kg raw beef and 2.9–5.4 kg CO_2_-eq/kg raw pork [23], where cuts with a higher price are associated with a higher CF [18]. Since the dietary intake data did not distinguish between different cuts of meat, we assigned all beef and all pork cuts in the intake data average CF values for beef and pork, as reported by CONCITO: 50.7 and 4.7 kg CO_2_-eq/kg beef and pork, respectively [135,136]. We estimated average iLUC values for beef and pork based on the different values provided in the BCD table: average iLUC 8.57 and 0.65 kg CO_2_-eq/kg for beef and pork, respectively. Similarly, we estimated average values for chicken based on a whole chicken and an unavoidable loss of 23%, as for the AU-DTU data. When no match was found for certain fish species, intake data were matched with a fish species in the BCD according to the product assumed to replace it in the market [18].

### 2.5. Estimation of CF from the Diets and Statistics

The CF from the total current diet was calculated on an individual level for all participants of the dietary survey by multiplying the CF of each food item and individual food intakes. The results are provided for the total population and for men and women separately, as means or medians, standard deviation (SD) or percentiles in kg CO_2_-eq per day and per 10 MJ. The 10th and 90th percentiles were calculated to present the range of distribution in CF in the population. The estimated CF for the plant-rich diet was calculated per 10 MJ from modelled average food intake amounts.

The changes in the CF from current to plant-rich diet were estimated at an individual level by assigning of the CF of the plant-rich to each individual and calculating the difference between the CF of current diet and the plant-rich per 10 MJ. The CF contributions from selected food groups were estimated for the current diet and the modelled plant-rich diet based on population averages of food intakes per 10 MJ for both diets.

The differences between men and women in terms of the CF of the current diet and the CF reductions were tested statistically with independent-samples t-tests, while the corresponding differences between CF data sets were tested statistically using paired-samples *t*-tests. Statistical significance was declared at *p* < 0.01.

All individual-level calculations, descriptive statistics and statistical tests were carried out with R statistical software (version 3.6.3), while calculations of the CF of the plant-rich diet and of food groups based on population averages were made using MS Excel spreadsheets.

## 3. Results

### 3.1. The CF of the Current Diet

Table 1 shows the CF of the current diet including CF contribution until the retail gate for the total adult population and for men and women separately using three different CF data sets (AU-DTU, BCD excl. iLUC and BCD incl. iLUC). Results for total CF at the household level are shown in Supplementary Materials Table S5. The results differed depending on the boundaries used (retail gate or household level) or whether results were expressed per person per day or per 10 MJ.

Based on the AU-DTU CF data set, the average CF of the current diet per person per day at the retail gate was 4.23 kg CO_2_-eq. The average CF was 33% higher among men than women, and the highest 90th percentile among men was 2.7-fold higher, on average, than the lowest 10th percentile among women. When the calculation was based on the CF per 10 MJ, no significant difference was found between the average CF of the diets of men and women (4.38 and 4.37 kg CO_2_-eq per 10 MJ, respectively).

Using the BCD data set excl. iLUC, the average estimated CF of the current adult diet per person per day was 10% higher than when using the AU-DTU data set, while it was 25% higher when using the BCD data set incl. iLUC. The same percentage differences were seen when expressed as CO_2_-eq per 10 MJ. The average estimated CF of the current diet was 41% and 43% higher, excl. and incl. iLUC, respectively, for men than women, measured as CF per person per day. The difference between the highest 90th percentile among men the 10th percentile lowest among women was also higher (3.7–3.8 fold). When measured as CF per 10 MJ, the average CF of the current diet was 7% and 8% higher among men than women for BCD excl. iLUC and incl. iLUC, respectively (Table 1).

The average estimated CF of the current diet at the household level was approximately 9% higher than at the retail gate, when using AU-DTU data, and 11% higher when measured by either BCD data excl. iLUC or incl. iLUC (see Supplementary Materials Table S5).

### 3.2. CF Reduction on Transition from Current to Plant-Rich Diet

The CF of the modelled plant-rich diet was estimated to be 3.01 kg CO_2_-eq per 10 MJ at the retail gate, using the AU-DTU data set. The same level was found when using BCD data incl. iLUC (3.04 kg CO_2_-eq per 10 MJ), whereas the use of BCD data excl. iLUC resulted in an approximately 10% lower value (2.72 CO_2_-eq per 10 MJ). Corresponding estimates at the household level were 3.32, 3.02 and 3.37 CO_2_-eq per 10 MJ (AU-DTU data, BCD excl. and incl. iLUC).

Calculation of the CF of the plant-rich diet (per 10 MJ), including food losses at the household level, using AU-DTU data was approximately 10% higher than at the retail gate for the plant-rich diet, as was the case for the current diet. When using BCD data, the CF at the household level was approximately 11% higher than at the retail gate. This means that household losses account for approximately 10% of the total CF at the household level.

Table 2 shows the estimated reduction in the CF of the transition from the current diet to the plant-rich diet. There was no difference in the CF reduction between men and women using AU-DTU data, but men had a significantly larger CF reduction when using BCD data. The CF reduction using AU-DTU data was significantly smaller compared to the reduction using BCD data, both including and excluding iLUC. The CF reduction was found to be 31% when using the AU-DTU data, while the estimated reduction was 43% and 44% when using BCD data excl. and incl. iLUC, respectively. Similar reductions were found when including household losses.

### 3.3. CF Contribution from Food Groups

**Figure 1:**
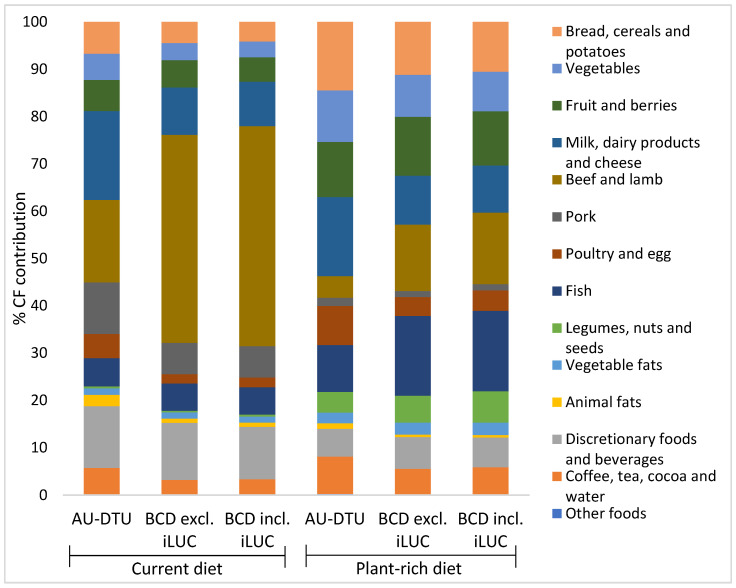
Proportion (%) of the total carbon footprint (CF) from selected food groups in the current diet and the plant-rich diet (10 MJ), and estimated based on three sets of CF data (AU-DTU and BCD +/− iLUC). System boundary is at the retail gate. The current food intake: The Danish National Survey on Dietary habits and physical Activity (DANSDA) 2011–2013, adults 18–64 years [24]. The food intake of the plant-rich diet: Lassen et al. 2020 [9]. The AU-DTU data are compiled by researchers from Aarhus University and DTU (Technical University of Denmark) described in methodological Section 2.3 of the present study. The Big Climate Database (BCD): published by the Danish green think tank CONCITO [23] described in [18]. iLUC: indirect land use change.

Table 3 shows the content in g per 10 MJ of foods aggregated into main food groups of the current diet and the plant-rich diet. Further, Table 3 shows the CF contributions of these food groups in kg CO_2_-eq per 10 MJ using the three different CF data sets. Figure 1 illustrates the relative contribution of each food group to the total CF using the different CF data sets.

In the current diet, the CF from animal products accounted for 61%, plant products (i.e., bread, cereals and potatoes (starchy foods), vegetables, fruits, legumes and nuts, and vegetable fat) for 21%, discretionary foods and beverages for 13% and other beverages for 6% when using AU-DTU data, while 69% and 71% came from animal products, 16% and 14% from products of plant origin, 12% and 11% from discretionary foods and beverages and 3% and 3% from other beverages, using BCD data excl. and incl. iLUC, respectively.

Using AU-DTU data, ruminant meat (beef and lamb) accounted for approximately 18% of the total CF of the current diet. Furthermore, other meat and eggs accounted for 16% and dairy and cheese accounted for 17%. Fish only accounted for 6% of the total CF of the current diet.

When using BCD data, ruminant meat (beef and lamb) accounted for approximately 45% of the total CF of the current diet. This was the case independently of whether iLUC was included. The contribution from other meat and eggs was approximately 9% and from dairy and cheese 10%. Fish also accounted for approximately 6% of the total CF.

With regard to the plant-rich diet, the CF from animal products accounted for 42%, plant products for 44%, discretionary foods and beverages for 7% and other beverages for 8% when using AU-DTU data, while 47% and 48% came from animal products, 41% and 40% from products of plant origin, 7% and 6% from discretionary foods and beverages and 5% and 6% from other beverages, using BCD data excl. and incl. iLUC, respectively.

Looking at the plant-rich diet and using AU-DTU data, the ruminant meat (beef and lamb) accounted for approximately 5% of the total CF. The contribution from other meat and eggs was 10% and from dairy and cheese was 17%. Fish accounted for 10% of the total CF of the plant-rich diet.

When using the BCD data, the picture was different, but again showing the same pattern independently of iLUC being included. The ruminant meat (beef and lamb) accounted for approximately 14–15% of the total CF of the plant-rich diet. The contribution from other meat and eggs was approximately 5.5% and from dairy and cheese 10%. Fish accounted for approximately 17%.

**Figure 2:**
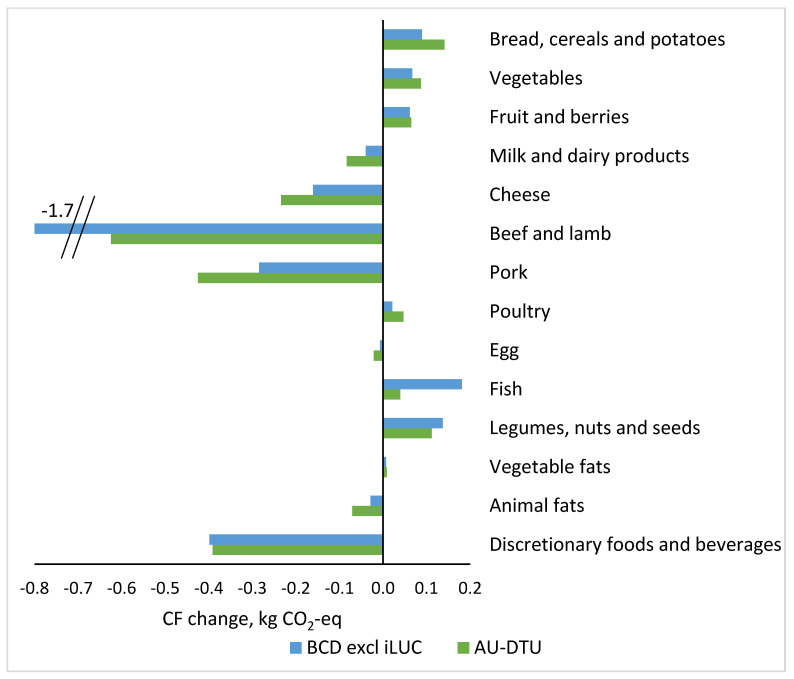
Contribution to the carbon footprint (CF) change in the transition from the current diet to the plant-rich diet from selected food groups and estimated based on two sets of CF data, kg CO_2_-eq per 10 MJ diets. System boundary is at the retail gate. The current food intake: The Danish National Survey on Dietary habits and physical Activity (DANSDA) 2011–2013, adults 18–64 years [24]. The food intake of the plant-rich diet: Lassen et al. 2020 [9]. The AU-DTU data are compiled by researchers from Aarhus University and DTU (Technical University of Denmark) described in methodological Section 2.3 of the present study. The Big Climate Database (BCD): published by the Danish green think tank CONCITO [23] described in [18]. iLUC: indirect land use change.

Figure 2 shows the contribution from selected food groups to the CF changes in kg CO_2_-eq in the transition from the current diet to the plant-rich diet comparing calculations with BCD CF data excl. iLUC with AU-DTU CF data (which also is excl. iLUC). The right side of the vertical line shows the food groups that contribute with an increased amount of CF in the plant-rich diet compared with the current diet, and the left side shows the food groups that contribute to decreased amounts of CF in the plant-rich diet. The sizes of the changes are dependent on both how much the food group has increased or decreased in amount and the CF of the foods per kg.

Figure 2 shows different results depending on which of the sets of CF data were used. The decrease in the CF from beef and lamb when using the BCD data was approximately 3-fold the decrease when using AU-DTU data, while the contribution to the reduction is less from pork and cheese. The decrease in the CF from discretionary foods and beverages is approximately the same. On the other hand, the increase in the CF from fish is higher when using BCD data.

## 4. Discussion

The present study compared two different approaches for estimating CF reduction on the transition from the current Danish diet to a plant-rich diet per 10 MJ. Using AU-DTU data, based on aLCA from existing literature values, estimated CF reduction was found to be 31%. When using BCD data, based on cLCA and a top-down hybrid approach, the estimated reduction was 43% and 45% excl. and incl. iLUC, respectively.

### 4.1. The Current Diet

The estimated CF of the current diet in the present study, i.e., the average adult diet from DANSDA 2011–2013, was 4.78 kg CO_2_-eq/10 MJ (including food waste at the household level). The CF was comparable to but a little higher than earlier results based on a previous Danish dietary survey from 2005 to 2008 [50]. The differences in CF are due to differences in food composition and updated CF values of individual foods in the present study. For example, beef content was higher in the present study than in the average 2005–2008 diet. The AU-DTU data set was an update of the data set used for calculations on the 2005–2008 intake patterns: using the same literature-based approach and existing LCA studies but updated and covering a more detailed food intake.

Using the SHARP indicator database (SHARP-ID), developed in the 5 year EU funded SUSFANS project and including GHG emissions and land use related to individual foods coded by FoodEx2 classification system from the European Food Safety Authority, the CF of the average adult Danish diet 2005–2008 was estimated to be 5.0 kg CO_2_-eq per 2000 kcal (approximately 6 kg CO_2_-eq/10 MJ) [137]. Although the SHARP-ID was based on aLCA studies (as the AU-DTU database), it included more global CF values, and for example CF of beef is twice the AU-DTU value.

Using the BCD data set, the CF of the average current diet was approximately 10% higher than the AU-DTU estimate and 25% higher when BCD data included iLUC, independently of whether estimated at the retail gate or the household level.

The estimated CF of the current diet in this study is comparable with the impact of self-selected diets estimated and reviewed from other studies by Heller et al. 2018 using different databases and type of food intake data (4.7–8.8 kg CO_2_-eq per capita per day, including consume and losses) [116], and the Swedish diet among adults above 56 year of age (5.5 CO_2_-eq per capita per day) [138].

### 4.2. Gender Differences

On average, the CF of the diet (estimated CF per day) is higher among men than among women, in accordance with the higher energy intake among men, which is also reported in other studies [137,138,139,140,141,142]. When energy adjusted to same energy level, there was no difference in CF of the diets of men and women in our study using AU-DTU data, in line with the other European studies [137,138,140]. Mertens et al. reported that Danish women had higher intake of fruits and vegetables and lower intake of red and processed meat than men [137]. This was also seen in the Danish diet from 2011 to 2013 in this study, where, in addition, women have a higher total intake of fish, poultry, eggs and cheese [24], which seems to even out the lower CF from red meat intake in women. Hallström et al. mention the higher content of meat in men’s diet and a higher content of discretionary foods contributing to levelling the energy-adjusted GHG emissions somewhat [138]. Similar to our results based on the BCD data, Sjörs et al., 2017 found lower CF of the diet of women (only 6%) compared with men [143], while Hjort et al., 2020 found a 20% difference [144]. Our results indicate that dietary changes are not only important among men due to their higher energy intake and meat intake but for both men and women to obtain the lower CF of transition to a plant-rich diet.

### 4.3. CF Reduction by Transitioning to a Plant-Rich Diet

The estimated CF of the plant-rich diet in the present study based on AU-DTU data set was 3.32 kg CO_2_-eq /10 MJ (including food waste at the household level) and 3.02 kg CO_2_-eq /10 MJ at the retail level, corresponding to a 31% reduction in the CF of the transition from the current diet to the plant-rich diet recommended by the official Danish FBDG for health and climate. Larger estimated CF reductions were found when calculations were based on BCD data: 43% and 44% excl. iLUC and incl. iLUC, respectively.

The estimated CF values of the plant-rich diet based on AU-DTU data are comparable to the estimated CF by Kovacs et al. of the recommended German and Dutch diets (both including sustainability considerations), when taking into account that calculations were based on 2000-kcal diets [145]. The CF database used by Kovacs et al. is based on an extensive literature review of LCA studies and global values; however, boundaries seem to be at the farm gate or the processor gate. Additionally, differences in food composition, e.g., regarding fish and meat, influence the total CF of the diets.

Other studies have compared the CF of current diets and diets following the national FBDGs. However, most of these studies covering Western countries are based on diets following FBDGs which do not include environmental sustainability or climate impact, and have found CF changes between +5% and −35% [11,12,146].

A few studies have compared the current diet with a modelled sustainable or climate-friendly diet. Martin and Brandão found that a reduced meat content of the Swedish diet could reduce the CF by approximately 20%, whereas vegetarian and vegan diets led to potential CF reductions of nearly 40% and 70%, respectively [147]. A diet consistent with the Eatwell Guide had a 32% lower environmental footprint than the current national diet in the UK [148], and a modelling study on average diets among UK adults demonstrated that diets conforming to WHO recommendations reduced the CF by 17% [149]. Further reductions of approximately 40% could be achieved by making realistic modifications to diets so that they contain fewer animal products and processed snacks and more fruit, vegetables and cereals. The effect of changing the current Dutch diet to a diet according to the Dutch FBDG results in CF changes from −13% for men aged 31–50 years to +5% for women aged 19–30 years [150]. Replacing meat in this diet and/or consuming only foods with relatively low GHG emissions resulted in average GHG emission reductions varying from 28 to 46%.

Household losses account for approximately 9% to 10% of the CF of both diets and independently of the data used in this study. Despite a relatively higher amount of household losses of plant-based foods compared to animal-based foods, the share of household food losses in the plant-rich diet CF was only slightly higher compared with the current diet. However, the contribution to GHG emissions from food waste is substantial. Food losses along the whole chain constitute 18% of the CF of the diet in a recent Swedish study [138], and approximately 8–10% of total anthropogenic GHG emissions is estimated to correspond to food loss and waste [4].

### 4.4. Food Group Contributions to CF Reduction

When using AU-DTU data, the CF reduction in the present study was mainly driven by the lower content of beef and lamb in the plant-rich diet, which accounted for a decrease in the CF of 0.63 kg CO_2_-eq/10 MJ, but also from the large decrease in the content of pork and discretionary foods and drinks, both CF reductions being approximately two-thirds of the reduction in CF from ruminant meat (Figure 2). A smaller amount of cheese also contributed with a substantial CF reduction with approximately two-fifths of the reduction in CF from ruminant meat. As illustrated in Figure 1, animal products constitute approximately 61% of the total CF of the current diet, and 42% of the plant-rich diet. The plant-based foods constitute 20% and 44% of the CF of the current and plant-rich diets, respectively, while the CF from discretionary foods and drinks constitute 13% and 7% and other drinks 6% and 8%, respectively. These results indicate that obtaining a lower CF from the diet requires a lower intake of beef and lamb but focus on a low intake of other types of meat and cheese, and discretionary foods and beverages is needed as well. From a nutritional point of view, an increase in fruit, vegetables, vegetable oils, and plant-based protein-rich foods such as legumes, nuts and seeds—as well as the small amounts of meat, and moderate amounts of fish and dairy—is important [9,13,14]. These results are in line with the review of Gonzales-Garcia et al. (2018) based on 21 peer-reviewed studies and comparison of nutritional scores and CF of 66 dietary scenarios [22].

When using BCD data, the lower content of beef and lamb accounted for a decrease in CF of 1.7 kg CO_2_-eq /10 MJ, which is 2.6-fold the reduction from beef and lamb in AU-DTU data (Figure 2). The CF from ruminant beef accounted for 45% of the CF from the current diet compared with 18% when using AU-DTU data, while being 15% versus 5% of total CF of the plant-rich diet (Figure 1). Discretionary foods and drinks accounted for a reduction approximately the same size with the two data sets but the reductions from the lower content of pork, cheese, milk and dairy products were smaller with BCD data compared to the results when using AU-DTU data (Figure 2). Compared with the results obtained with AU-DTU data, these data show that a lower content of beef and lamb is the most important change in the diet, whereas the contribution to the reduction from the other changes is considerably less important. The CF value of beef in the AU-DTU data set is low compared to other data on beef [21,73] because of a relatively high proportion of dairy beef, representing beef in the Danish market [50]. The average CF value of beef in the BCD data set is 3.5-fold higher than in the AU-DTU data. This large difference is due to the BCD using cLCA and system expansion where CF of beef is based on imported beef breed cattle [18]. At the same time, this approach with system expansion provides lower CF values of dairy products, including cheese and butter. This is also the main reason for the higher CF reduction on the transition from the current to plant-rich diet when using BCD data.

On using the AU-DTU data, other studies have also pointed out several food changing strategies including, but not limited to, balancing energy intake and lowering beef intake [140].

### 4.5. Strengths and Limitations of the Present Study

The direct comparison of two data sets of CF values adjusted to match food consumption data using the same system boundaries is a major strength of this study. Furthermore, intake data are based on dietary intake surveys from a representative sample of the Danish population (the DANSDA survey) and therefore reflect the average food preferences and eating habits of the population [24]. The data constitute a high level of detail with regard to foods in each food group. A limitation is that the data are approximately ten years old; but on average, the data are considered to represent the Danish current diet fairly well because dietary changes at a population level are generally slow. However, it is possible that changes towards lower milk, lower or/and altered meat intake, and slightly higher intake of legumes have occurred since the collection of data. Energy adjustments of food intake per 10 MJ account for the under-reporting often seen in dietary surveys among adults [151]; however, under-reporting may be specific to certain food groups, which could not be adjusted for in the present study. In addition, another limitation is that the results do not take the potential CF impact of overconsumption into account. Due to inherent problems with under-reporting in dietary assessments, the energy surplus in the population that leads to an increasing prevalence of overweight individuals is not quantified. CF reductions from balancing energy intake and expenditure have previously been estimated to 0–10% [12,139]. This is in line with Hall et al., 2011, who estimated that maintaining the obesity epidemic in the US requires a daily energy surplus of 0.9 MJ [152] and with Mertens et al. 2019 [137], who concluded that lowering energy intake without changing diet composition, i.e., proportionally lowering intake from each food group, would be one strategy for reducing GHG emissions and land use of the daily diet. Therefore, the estimated CF of the current diet per person per day may be up to approximately 10% too low and the combined effect of the transition to the plant-rich diet and a balancing energy intake would be more than the estimated 31% CF reduction. In contrast, an increase in physical activity level is also recommended and this will increase energy expenditure to some extent.

The plant-rich diet directed at those aged 6–65 years was represented by only one scenario, although sustainable and healthy plant-rich diets might be achieved in multiple ways. Future research should investigate the impact of choosing different sets of CF data on the most sustainable food patterns, including more scenarios across food groups and within food groups as well as the impact of introducing new protein-rich plant-based products and plant-based beverages. The current modelled plant-rich diet includes the same amount of coffee and tea as the current dietary patterns. A reduction in the amount of coffee and tea does not need a substitution with other foods/drinks (except perhaps tap water) and would further reduce the CF of the plant-rich diet compared with the current diet. In addition, other combinations of foods within the food groups could influence the CF of the diet, for example changes in the combination of fish to a larger share of seafood with lower CF in the AU-DTU data set, as recommended in the FBDG.

### 4.6. Data Uncertainties

There are uncertainties related to both sets of CF data. The CF for a given food type can vary widely by production system and within regions, and depends on the choice of standard factors used in both approaches. The AU-DTU data are based on availability of LCA studies. For certain products, this is scarce, for example for processed and composite foods, where the uncertainties in CF data of these foods are greater. However, for most food commodities where consumption is considerable, the literature is more abundant, thereby limiting the overall uncertainty. Exceptions are vegetables both grown in greenhouses and on free land, e.g., tomato and cucumber, where the estimated values are possibly low in the AU-DTU data.

The CF for chicken and pork are at the same level in the AU-DTU data. Future research should include new LCA studies for Danish chicken production and may well provide a lower CF, which is in line with newer CF data on pork (e.g., Moberg et al. [39]). In addition, the standard values and estimated values used for packaging, processing and transport are the best estimates at present, but contribute to uncertainties, and should be further explored.

The contributions of industrial food processing (such as grinding, cutting, centrifuging and washing), storage, and transport from retail to home have previously been estimated to contribute up to 32% to the environmental impact for highly processed foods such as pizza, as mentioned by Mertens et al. [137]. Further, estimates have shown an increasingly important role of food-related emissions generated outside of agricultural land, in pre- and post-production processes along food supply chains (manufacturing of fertilizers, food processing, packaging, transport, retail, household consumption and food waste disposal), now accounting for 35% [153], suggesting that more attention should be paid to correct estimations in future LCA studies. Tubiello et al. conclude that this has important repercussions for food-relevant national mitigation strategies, considering that until recently these have focused mainly on reductions in non-CO_2_ gases at the farm and on CO_2_ mitigation from land use change. In particular, Seferidi et al. highlight that current evaluations of the environmental impacts of diets fail to adequately address the environmental impacts of ultra-processed foods [154]. This suggests an overall underestimation of the CF of both the current and the plant-rich diets in this study, but probably not the estimated CF reduction, as only small (and similar) amounts of processed foods are included in both diets.

### 4.7. LCA Methodology

The two CF data sets used in this study have fundamentally different approaches. One might expect a higher CF of the total diet using data provided by the top-down hybrid approach than by the bottom-up approach, and we could expect higher CF values of the individual foods, since the top-down approach should include all GHG emissions and break down to food sectors, through food producers and finally the CF of food items [18].

In the present study, as expected, the CF was higher for the average current diet when estimated by the BCD (top-down) than the AU-DTU data (bottom- up): 4.79 versus 4.37 CO_2_-eq/10 MJ (at the retail gate). BCD including iLUC was 5.46 CO_2_-eq/10 MJ. However, this was not the case for the plant-rich diet, where the calculated CF was 2.72, 3.01 and 3.04 CO_2_-eq/10 MJ by BCD data excl. iLUC, AU-DTU data (also excl. iLUC) and BCD incl. iLUC, respectively. A study by Sugimoto et al., 2020 investigated the impact of using literature-based data (bottom-up), and two types of top-down data: production-based IOT and consumption-based IOT methods, the latter using prices at the consumer level in data compilation [20]. Similar to this study, the CF of the current Japanese diet was higher when using consumption-based IOT data. However, beef accounted for a lower proportion of total CF when using consumption-based IOT data than using literature based data (bottom-up)-probably due to different dietary habits and relative food prices in Japan than in Denmark.

By aligning the system boundaries of both data sets to fit dietary data, we have attempted to make data comparable and the CF representative of real-life practice. Still, there is a risk of systematic differences between data. For example, for AU-DTU data, avoidable food waste in retail is included, which results in approximately 5% higher total CF than if losses in retail were not included. It is assumed that losses in production and retail are included in BCD data. In addition, the contribution from storage, which is estimated as overall for retail and household, may account for a systematically higher CF of the diets calculated with AU-DTU data. Looking at the CF of individual foods, values in the BCD are higher for some foods such as beef, some fishes (especially herring and mackerel) and pulses. In particular, the CF of beef was much higher (3.5 fold) in BCD data. Other foods such as pork, poultry, eggs, some fishes (e.g., flatfish and shellfish), milk, cheese, butter, bread and flour, and many vegetables had a lower CF in the BCD data.

### 4.8. Future Perspective

FBDG are promoted in countries all over the world in order to improve health and reduce the risk of chronic diseases, and new FBDGs which include sustainability considerations are emerging in more and more countries [155] as recommended by the FAO/WHO [156]. Additionally, the awareness of the importance of and need for urgent and big changes in the food system is increasing [157,158]. A study from 2020 concluded that changes in food consumption pattern is needed, but also that the change from the production side is imperative. Efforts from the production side, e.g., enhancing yield and improving production efficiency, are encouraged to further lower environmental footprint [159]. Future changes in food consumption patterns and production system changes, including using technological advances and the new improved production systems, are needed and foreseen. Barisan et al. argue that the adoption of practices aimed at reducing the carbon footprint also allows the creation of economic benefits for firms as well as for the environment [160]. The possible effects of these future developments on the CF of the individual food items are not reflected in the calculations in the present study, where the estimated CF of the current and the modelled diets are based on present-day (and past) CF values based on data of the present production systems. Therefore, continuously updating the CF of food and food systems is needed.

This study has focused on GHG emissions and the climate impact; however, studies have shown that food consumption also impacts other sustainability parameters, as included in the 17 Sustainable Development Goals, launched in 2015 by UN [161]. A study by Moberg et al., 2020 showed that the environmental impacts caused by the average Swedish diet exceeded the global boundaries for greenhouse gas emissions, cropland use and application of nutrients by 2 to more than 4 fold when the boundaries were scaled to a per capita level [85]. With regard to biodiversity, the impacts caused by the Swedish diet transgressed the boundary by 6 fold. For freshwater use, the diet performed well within the boundary. Although the overall picture reveals that the impact on most parameters is driven by meat and other animal-based food products, except for water depletion, differences might appear when investigating the impact of different food items within each food group [54,85]. Using a macro-scale top down approach, a study on EU food consumption based on 28 EU member states identified food products, in particular meat and dairy products, as key contributors to acidification, eutrophication, land use, and water use, and to a lower extent climate change [162]. Finally, several manufactured products, raw materials and basic products, contributed significantly to impacts on human toxicity, freshwater ecotoxicity and resource uses [162]. These results indicate that there is a need to establish data for all impact categories in the future, for each food item to avoid potential pollution swapping by focusing only on one impact category, e.g., climate change [15].

Recommendation “on the use of the Environmental Footprint methods to measure and communicate the life cycle environmental performance of products and organisations” from EU Commission 2021 (incl. annex1–3) is based on several years of work on development of Product Environmental Footprint Category Rules (PEFCRs) [17]. These PEFCRs aim at promoting standardization of the LCA methods used, e.g., they recommend use of aLCA studies and that dLUC can be included if reported separately, but iLUC cannot be included due to excessive uncertainty in the method. PEFCRs include a high number of impact categories such as climate change, ozone depletion, human toxicity (cancer and non-cancer), photochemical ozone formation (human health), acidification, eutrophication, terrestrial, freshwater and marine, ecotoxicity (freshwater), land use, and water use. So far, biodiversity impacts are not included in PEFCRs. Others have highlighted that the aLCA methodology is considered more suitable for use in policy decisions with regard to changed food consumption patterns [163]. Additionally, Moberg et al. made use of an aLCA approach in a study on climate-tax models, although cLCA accounts for emissions caused by producing a specific food item on the margin, which could be considered more in line with economic theory on taxing marginal emissions. However, it was considered that such an approach depends on many assumptions and is therefore highly uncertain, difficult for non-experts to interpret and costly to update [39].

The plurality of perspectives to achieving transformation in food systems and the need to take all of these environmental sustainability metrics into account to obtain a clean and healthy planet, in addition to ensuring healthy, nutritional adequate and safe dietary changes; just, ethical and equitable food systems; and economically thriving, robust food value chain call for the urgency of managing the trade-offs as discussed and substantiated in a study by Hebinck et al. [158]. They describe a framework that provides the foundation for the design of integrated policies that reflexively manage trade-offs throughout the various stages of policy processes to foster multi-actor, inclusive negotiations and ensure reflexive and comprehensive evaluations. They also conclude that the implementation of the framework hinges on availability of data and consensus on policy targets [158]. The AU-DTU CF data in the present study are intended for use in the calculation of the CF of whole diets but are not suited for CF labelling of specific food products and can only, on an overall level, be used to prioritize between individual foods within food groups when planning food purchases.

However, results can provide the basis for reflection on guidelines and advice that can promote the desired transformation towards a sustainable healthy diet and the impact of using different databases of CF values.

## 5. Conclusions

This study provides insight into the CF of the current diet as well as the potential CF reduction through transitioning to a diet consistent with the Danish climate-friendly dietary guidelines depending on different sources of CF data. The transition from the current diet to the plant-rich diet showed a substantial estimated CF reduction of 31% with AU-DTU data, and a greater reduction with BCD data (43%). Ruminant meat reduction was the largest contributor to this CF reduction, especially with the use of BCD data, and other animal-based foods also contribute considerably to the CF reduction, especially with AU-DTU data. This study demonstrates that the choice of data is important when assessing both total CF of the diets and the relative contribution to CF of different food groups. Careful consideration of the source of CF data is crucial for dietary guideline development in order to promote both improved health and climate reduction. Further research is needed to be able to implement the transition to sustainable dietary habits and food system changes. Additionally, continuously updated market-specific data on the sustainability footprint based on standardized methodology are needed. Collaboration and co-creation between stakeholders, and development of tools that can guide consumers, and also producers and food and health authorities, are needed. This includes real-life studies involving consumers and producers in the implementation of dietary changes.

## Figures and Tables

**Table 1:** Total carbon footprint (CF) of the current diet among adults (18–64 years), per person per day and per 10 MJ, respectively #, calculated using three different CF data sets at the retail gate.

	CF, AU-DTU Data			CF, BCD Excl. iLUC			CF, BCD Incl. iLUC		
	Average	Men	Women	Average	Men	Women	Average	Men	Women
	*N =* 2492	*N =* 1202	*N =* 1290	*N =* 2492	*N =* 1202	*N =* 1209	*N =* 2492	*N* = 1202	*N* = 1290
**CF kg CO_2_-eq/pers/day**									
Mean	4.23 ^a^	4.85	3.65 ***	4.63 ^b^	5.47	3.86 ***	5.28 ^c^	6.26	4.38 ***
(SD)	(1.40)	(1.43)	(1.10)	(2.06)	(2.19)	(1.57)	(2.43)	(2.59)	(1.86)
Median	4.03	4.72	3.54	4.23	5.07	3.58	4.80	5.78	4.05
(P10;P90)	(2.70; 6.02)	(3.24; 6.59)	(2.45; 4.90)	(2.50; 7.31)	(3.12; 8.29)	(2.25; 5.67)	(2.78; 8.43)	(3.49; 9.55)	(2.50; 6.49)
**CF kg CO_2_-eq/10 MJ**									
Mean	4.37 ^a^	4.38	4.37	4.79 ^b^	4.97	4.63 ***	5.46 ^c^	5.69	5.25 ***
(SD)	(0.86)	(0.82)	(0.90)	(1.76)	(1.76)	(1.76)	(2.12)	(2.11)	(2.11)
Median	4.24	4.26	4.22	4.45	4.61	4.27	5.05	5.26	4.82
(P10:P90)	(3.42; 5.50)	(3.45; 5.40)	(3.38; 5.56)	(3.06; 6.92)	(3.17; 7.23)	(2.98; 6.61)	(3.40; 7.99)	(3.54; 8.44)	(3.30; 7.65)

# Average energy intake per day for the entire adult population aged 18–64 years is 9.81 MJ (3.12 MJ), and 11.24 MJ (3.24 MJ) and 8.49 MJ (2.32 MJ) for men and women, respectively (SD, standard deviation, in brackets). Significant difference between results of the total population in the same row is indicated with different letters ^a^, ^b^, ^c^ (*p* < 0.01). Significant difference between gender is indicated: *** = *p* < 0.001. The current food intake: The Danish National Survey on Dietary habits and physical Activity (DANSDA) 2011–2013, adults 18–64 years [24]. The AU-DTU data are compiled by researchers from Aarhus University and DTU (Technical University of Denmark) described in methodological Section 2.3 of the present study. The Big Climate Database (BCD): published by the Danish green think tank CONCITO [23] described in [18]. iLUC: indirect land use change.

**Table 2:** The carbon footprint (CF) change from current to plant-rich diet at the retail gate (kg CO_2_-eq per 10 MJ) calculated using three different CF data sets.

	CF, AU-DTU Data			CF, BCD Excl. iLUC			CF, BCD Incl. iLUC		
	Average	Men	Women	Average	Men	Women	Average	Men	Women
	*N* = 2492	*N* = 1202	*N =* 1290	*N =* 2492	*N =* 1202	*N =* 1209	*N =* 2492	*N* = 1202	*N =* 1209
**CF change** **kg CO_2_-eq/10MJ**									
Mean	−1.36 ^a^	−1.37	−1.36	−2.07 ^b^	−2.25	−1.90 ***	−2.43 ^c^	−2.65	−2.22 ***
(SD)	(0.86)	(0.82)	(0.90)	(1.76)	(1.76)	(1.76)	(2.12)	(2.11)	(2.11)
Median	−1.23	−1.25	−1.21	−1.72	−1.89	−1.55	−2.01	−2.22	−1.78
Mean change	−31%	−31%	−31%	−43%	−45%	−41%	−44%	−47%	−42%

Significant differences between results of the total population in the same row are indicated with different letters ^a^, ^b^, ^c^ (*p* < 0.01). Significant differences between gender are indicated: *** = *p* < 0.001. The current food intake: The Danish National Survey on Dietary habits and physical Activity (DANSDA) 2011–2013, adults 18–64 years [24]. The food intake of the plant-rich diet: Lassen et al. 2020 [9]. The AU-DTU data are compiled by researchers from Aarhus University and DTU (Technical University of Denmark) described in methodological Section 2.3 of the present study. The Big Climate Database (BCD): published by the Danish green think tank CONCITO [23] described in [18]. iLUC: indirect land use change.

**Table 3:** Intake g/10 MJ and the carbon footprint (CF) (incl. waste at the retail gate and incl. cooking) of food groups in the current diet and plant-rich diet per 10 MJ based on three CF data sets. The amounts of food are primarily as raw weight, except for prepared foods as bread, sausages, cold cuts and smoked or canned fish products.

Food Group	Current Diet per 10 MJ				Plant-Rich Diet per 10 MJ			
		CF AU-DTU	CF BCD, Excl. iLUC	CF BCD, Incl. iLUC		CF AU-DTU	CF BCD, Excl. iLUC	CF BCD, Incl. iLUC
	g/10MJ	kg CO_2_-eq	kg CO_2_-eq	kg CO_2_-eq	g/10MJ	kg CO_2_-eq	kg CO_2_-eq	kg CO_2_-eq
**Bread and cereals**	195	0.25	0.16	0.17	306	0.38	0.24	0.25
**Potatoes**	85	0.05	0.06	0.06	100	0.06	0.07	0.07
**Vegetables, all**	226	0.24	0.17	0.18	307	0.33	0.24	0.25
**Fruit and berries, all**	243	0.29	0.28	0.28	303	0.35	0.34	0.35
**Milk and dairy products**	315	0.40	0.19	0.21	250	0.32	0.15	0.16
Milk	280	0.31	0.14	0.15	222	0.24	0.11	0.12
Other dairy	35	0.09	0.05	0.05	28	0.07	0.04	0.04
**Cheese**	45	0.42	0.29	0.31	20	0.19	0.13	0.14
**Meat, total**	168	1.40	2.51	3.00	56	0.40	0.51	0.62
Beef and lamb	52	0.76	2.12	2.55	9	0.14	0.38	0.46
Pork	87	0.48	0.32	0.36	9	0.05	0.03	0.04
Poultry	29	0.16	0.07	0.09	38	0.21	0.10	0.12
**Egg**	22	0.06	0.02	0.02	15	0.04	0.01	0.02
**Fish**	36	0.26	0.28	0.32	63	0.30	0.46	0.52
**Legumes, dry weight**	1	0.00	0.00	0.00	40	0.03	0.06	0.08
**Nuts and seeds**	6	0.02	0.02	0.02	38	0.10	0.09	0.12
**Vegetable fats**	23	0.06	0.06	0.07	25	0.07	0.07	0.08
**Animal fats**	12	0.11	0.04	0.05	4	0.03	0.01	0.02
**Discretionary foods and** **beverages**	518	0.57	0.58	0.61	157	0.18	0.18	0.19
**Coffee, tea, cocoa and water**	1987	0.24	0.15	0.18	1946	0.24	0.15	0.17
Other foods *	4	0.01	0.01	0.01	3	0.01	0.01	0.01

* Includes miscellaneous food consumed in limited amounts, e.g., spices. The current food intake: The Danish National Survey on Dietary habits and physical Activity (DANSDA) 2011–2013, adults 18–64 years [24]. The food intake of the plant-rich diet: Lassen et al. 2020 [9]. The AU-DTU data are compiled by researchers from Aarhus University and DTU (Technical University of Denmark) described in methodological Section 2.3 of the present study. The Big Climate Database (BCD): published by the Danish green think tank CONCITO [23] described in [18]. iLUC: indirect land use change.

## Data Availability

In accordance with Danish law, the confidential intake data used in this study can only be accessed through the servers at The Technical University of Denmark. Access to data not covered by supplemental material is granted upon request if the applicant fulfils the criteria for access. Then, data are available on request from the corresponding author.

## Supplementary Materials

The following are available online at https://www.mdpi.com/article/10.3390/foods11081119/s1, Table S1: Carbon Footprint (kg CO_2_-eq/kg) from primary production (farming) and processing of foods. When several references are given, the CF is the arithmetic mean of the different source data, unless otherwise indicated; Table S2: CF (kg CO_2_-eq/kg) related to packaging of different foods; Table S3: The CF (kg CO_2_-eq) from cooking 1 kg of raw food; Table S4: Estimated CF (kg CO_2_-eq/kg) related to storage in supermarket and home; Table S5: Total carbon footprint (CF) of the current diet among adults (18-64 year), per person per day and per 10 MJ, respectively*, calculated using three different CF data sets at household level.
