# Supplementary material for: Carbon Footprint Reduction by Transitioning to a Diet Consistent with the Danish Climate-Friendly Dietary Guidelines: A Comparison of Different Carbon Footprint Databases
Source: Foods. 2022 Apr 13;11(8):1119. doi: 10.3390/foods11081119 (PMC9030092; doi:10.3390/foods11081119)
Supplement: Supplementary file 1 [file foods-11-01119-s001.zip › foods-1641211-supplementary.pdf]

Supplementary material for

Trolle et al. Carbon Footprint Reduction by Transitioning to a Diet consistent with the Danish Climate-Friendly Dietary Guidelines: A Comparison of Different Carbon Footprint Databases. Foods, 2022.

Table of Contents

Table S1 Primary production and processing 2

Table S2 Packagning 6

Table S3 Cooking 7

Table S4 Storage 8

Table S5 Total CF of the current diet at household level 9

References 10

Table S1. Carbon Footprint (kg CO_2-eq/kg) from primary production (farming) and processing of foods. When several references are given, the CF is the arithmetic mean of the different source data, unless otherwise indicated.

Food group	Food item	Farm + Processing, kg CO_2-eq/kg	Reference
Meat and egg	Pork, raw [1]	4.25 [2]	[1,2]
	Beef and veal, raw [1]	12.88	[3] based on [4]
	Chicken, raw	2.84 [3]	[2] [4]
	Lamb, raw	27.91 [5]	[5]
	Processed pork products [6]	5.53	Calculated [7]
	Processed beef products [8]	16.32	Calculated [7]
	Game meat [9]	0	Estimate
	Egg	1.96	[6] [10]
Cereals and bread	Wheat flour	1.01	[6]
	Rye flour	0.98	[6]
	Wheat bread [11]	0.78	[6]
	Rye bread [12]	0.72	[6,7]
	Oats	0.66	[6]
	Pasta, dry	1.23 [13]	[8]
	Rice, dry	2.90	[9]
	Breakfast cereals	2.21	[10]
	Pastries, cakes and cookies	1.06	[11]
Milk, dairy and cheese	Skimmed milk	0.82	[12]
	Semi skimmed milk	0.88	[12]
	Whole milk	1.05	[12]
	Yoghurt	1.16	[12]
	Creme fraiche/cream (9-18% fat)	2.39–2.64	[12]
	Creme fraiche/cream (38% fat)	4.65–5.12	[12]
	Mould cheese	7.95	[12]
	Yellow cheese	8.66–9.47	[12]
	White cheese	6.93	[12]
	Cream cheese	6.11	[12]
	Cottage cheese	3.04	[12]
	Ice cream	3.78	[2]
Vegetables	Potatoes	0.15	[2,13–15]
	Broccoli	0.42	[2,15–18] [14]
	Tomato	0.69 [15]	[19–21]
	Carrot	0.14 [16]	[22]
	Other roots	0.15	[15,16,23–26]
	Spinach	0.20	[18] [14]
	Cucumber	0.47 [17]	[15]
	Salad/lettuce	0.42 [17]	[15,27,28]
	Bell pepper	0.98	[18,29,30] [14]
	Onion [18]	0.14	[22]
	Cabbage and kale [19]	0.18	[15,16,23,24,29]
	Green pea	0.43 [20]	[29,31,32]
	Corn, kernels	0.40 [21]	[33–35]
	Cauliflower	0.30	[2,18,36] [14]
	Leek	0.14	[16]
	Avocado	0.70 [21]	[18,29] [14]
	Olive	0.55	[37–39]
	Green beans	0.40 [21]	[17,24,29,33,36,40]

	Asparagus (green and white)	1.80 [21]	[18,29,33,41] [14]
	Pumpkin and zucchini	0.15	[24,42]
	Aubergine	1.35	[18,29] [14]
	Artichoke	0.50	[29]
	Jerusalem artichoke	0.30	[23]
	Champignon	2.80	[36,43–45]
Fruits	Apple	0.20 [21]	[2,13,15,46,47]
	Pear	0.20 [21]	[2,13,18,48] [14]
	Banana and mango	0.50 [21]	[36,39,49]
	Peach and nectarine	0.20 [21]	[13,29,35,50,51]
	Melon, all	1.00 [21,22]	[18,24,29,30,52] [14]
	Orange	0.20 [21]	[15,29,49,53–56]
	Tangerine	0.30 [21]	[18,29,51,57] [14]
	Lemon and grapefruit	0.25 [21]	[2,25,29,53]
	Grape [23]	0.50 [21,24]	[33,35,47,58–61]
	Pineapple	0.31	[18,36,62–64] [14]
	Kiwi	0.30 [21]	[65–67]
	Plum	0.32	[29]
	Apricot	0.38	[29,68]
	Cherry	0.40 [21,25]	[15,29,69,70]
	Fig	0.25 [21]	[29,33]
	Berries, all	0.70 [21]	[2,15,29,36,47,71–75] [26]
	Jam/marmalade, all types	0.90	Calculated from recipe [27]
Legumes, nuts [28] and seeds [29]	Beans, unspecified, dry [30]	0.24 [31]	[76]
	Soy beans, dry	0.29	[26]
	Chickpeas and lentils	0.25 [21]	[33]
	Split peas	0.35 [21]	[29,31,32] [32]
	Tofu	1.32	[77,78]
	Seeds, all	1.57	As sunflower seeds from [26]
	Peanuts	1.60	[79,80]
	Hazelnut	2.21	[29,80,81]
	Almond	3.45	[82]
	Walnut	2.34	[47,78]
Fish, mollusks and crustaceans [33]	Flatfish (plaice, halibut, flounder), raw	19.31	[2]
	Mackerel, garfish, raw	0.45	[2]
	Mackerel, smoked/in tomato sauce	0.67	Calculated from [2] [34]
	Salmon, farmed, raw	4.13	[2]
	Salmon, smoked	5.27	Calculated from [2] [34]
	Herring, raw	0.62	[2]
	Herring, pickled	1.01 [21]	[2,83]
	Tuna, canned [35]	2.15	[5,84]
	Rainbow trout	4.20	[2]
	Cod, raw	5.77	[2]
	Cod roe	1.28	[2]
	Saithe (Pollock)	3.80	[2]
	Mussel, raw	1.24	[85]
	Shrimp, frozen	11.85	[2]
	Crab claws	7.14	[86]
	Lobster	31.40	[87]

Fats and fat products	Margarines	1.60	[2,88,89]
	Mayonnaise, dressing etc.	2.04	Calculated [36]
	Rapeseed oil [37]	2.12	[26,90,91]
	Olive oil	3.66	[90,91]
	Sunflower oil	3.21	[26,90,91]
	Palm oil	4.23	[26]
	Palm kernel oil	5.65	[26]
	Soybean oil	1.14	[26,90,91]
	Coconut oil	2.73	[90]
	Butter	10.10	[12]
	Butter blends	6.46	[12]
Beverages, discretionary foods and other	Sugar	0.60	[26,92]
	Honey	0.92	[93]
	Candy/sweets	2.50	[94]
	Soft drink (soda/carbonated water)	0.18 [21]	[2,95]
	Milk chocolate	5.55	[2]
	Dark chocolate	3.90	[92]
	Coffee beans [38]	3.99	[2]
	Beer	0.18	[2] [39]
	Wine	1.44	[96]
	Spirits	1.00	[96]
	Water, tap	0.001	Estimated
	Juice, orange	1.32	[2]
	Juice, apple	0.53	[2]
	Juice tomato/vegetable	0.69	[97]
	Soy milk	0.47	[79]
	Nut butter with cocoa	1.94	Estimated [40]
	Peanut butter	1.80	Estimated [41]
	Chips, potato	2.48	[2](modified)
	Spices/condiments	0.50 [21]	[29](modified)

[1] All cuts, including edible offal. Edible boneless weight.
[2] CF corresponding to bone free meat. Obtained from 3.4 kg CO_2-eq/kg carcass (mean between the cited studies) with 1.25 as meat conversion factor from carcass to bone free meat [98]
[3] CF corresponding to carcass weight. 23% unavoidable loss [5] is deducted to obtain edible bone free meat.
[4] Values from Moberg et al., 2019 [2] are adjusted for Danish import.
[5] Global average. System boundary: regional distribution center.
[6] Incl. sausages, ham etc.
[7] Calculated from raw pork and beef, respectively, by adding CF for cooking (0.216 kg CO_2-eq/kg food) and 20% weight loss from cooking.
[8] Incl. cold cuts of beef.
[9] Incl. pheasant and hare, which are consumed in limited amounts.
[10] Value accessed through SimaPro in 2008.
[11] CF for wheat bread with seeds is estimated assuming 5% of the weight is sunflower seed.
[12] CF for rye bread with seeds is estimated assuming 15% of the weight is sunflower seed.
[13] Estimated as mix between dry and fresh pasta and wheat and durum flour.
[14] Stoessel et al., 20112 [18] and Jungbluth et al., 2016 [41] cited through Potter et al., 2020 [33].
[15] Estimated as 20% imported field grown and 80% greenhouse-grown.
[16] Average between two different storage systems: cold store or under straw matting in field.
[17] Literature values for cucumber and lettuce vary widely depending on production system and heat source assumed for greenhouse-grown products. Estimated as a mix between greenhouse-grown (with high proportion of renewable energy) and field grown.
[18] Spring onion estimated as x5 onion since inputs are similar but output lower due to earlier harvesting.
[19] All cabbages (red, spring) and kale are assigned the value for white cabbage.
[20] CF corresponding to green pea without pod. An estimated 0.125 kg CO_2-eq/kg for processing has been added for processing (removal of pod), based on Landquist & Woodhouse, 2015 [23] for processing of vegetables (without freezing).
[21] Estimate based on cited references.

[22] Represents mix between greenhouse-grown (CF 1.2 -1.5 kg CO_2-eq/kg) and field grown (0.30 kg CO_2-eq/kg).
[23] CF for raisin estimated as 5 x grape based on dry matter content.
[24] Estimate at the higher end of values found in literature based on Potter et al., 2020 [33] estimate for grapes in Swedish retail and because table grapes are estimated to have higher CF than grapes for wine production.
[25] Estimated at the higher end of vlaues found in literature since cherries are sometimes transported by air freight [33].
[26] Literature based on strawberries, blueberries and raspberries.
[27] Estimated 50% berries and 50% sugar with 0.25 kg CO_2-eq/kg for processing.
[28] CF of nuts estimated without shell. Edible fractions are 0.38 for hazel nuts, 0.69 for peanuts and 0.59 for almonds [33,99]. Walnut is assumed without shell in literature.
[29] Standard CF value 0.05 kg CO_2-eq/kg is added as estimated processing for all dry legumes, nuts and seeds.
[30] Incl. brown and white beans.
[31] CF for for dry beans calculated from CF for fresh beans based on dry matter content, assuming that 1.08 kg of fresh beans need to be cultivated for 1 kg of dry beans.
[32] CF from farming estimated from green peas.
[33] CF for all fish represents edible part since inedible parts are assumed removed at processing stage.
[34] Calculated from the raw fish by adding CF for cooking (0.108 kg CO_2-eq/kg food) and 20% weight loss from cooking.
[35] Edible part without CF from the packaging material.
[36] Calculated rapseed oil 63%, sunflower oil 15% and egg 6%.
[37] CF for grapeseed oil estimated as rapeseed oil.
[38] It is estimated that 45 g of coffee beans is needed for 1 litre of coffee ready to drink.
[39] Based to values in the Danish market: from Moberg 2019 [2] 80% Danish and 20% rest of the world (0.175 kg CO_2-eq/kg)
[40] 55%sugar+ +13%0palmoil+13%hazelnut+7% skimmilkpowder+7%cocoapowder+5% other (procesing:0.08kg CO_2-eq/kg food)
[41] As peanut + extra processing.

Table S2. CF (kg CO_2-eq/kg) related to packaging of different foods.

Type of packaging	Food item	CF, kg CO_2-eq/kg food	Reference
Paper/carton	Flour, grain, sugar	0.030	[100]
Carton + plastic cap	Cream (0.5 liter)	0.030	[12]
Plastic foil	Yellow cheese (800 g)	0.030	[12]
Carton + plastic cap	Milk, yoghurt (1 liter)	0.040	[12]
Plastic bag/paper/ carton	Rice, breakfast cereals, bread, biscuits, vegetables and fruit (fresh)	0.050	[100]
Plastic bag	Pasta, legumes (dried), nuts, seeds, fruit (dried), vegetables and fruit (frozen), candy	0.070	[100]
Paper carton + film	Mould cheese (150 g)	0.080	[12]
Carton + plastic	Mushroom (raw)	0.100	[100]
Plastic bag/ retort pouch	Snacks, potato chips	0.100	[101,102]
Plastic, modified-atmospheric packaging	Frozen fish	0.102	[102]
50% carton/50% plastic	Ice cream	0.121	[100,101]
Retort pouch	Coffee, tea	0.129	[101]
Carton	Eggs	0.145	[102]
Plastic bottle/ cans(steel)/glass bottle	Soft drinks, beer, juice	0.150	[2,96] [1]
Plastic tray + absorption pad + lid	Meat, cold cut, fish, tofu	0.160	[103,104]
Plastic pot	Shrimp in brine	0.211	[101]
Metal can/carton	Vegetables, preserved (tomato)	0.252	[105]
Plastic bag/ glass	Spices, dried tomato	0.260	[100,101,105]
Plastic tub	Cottage cheese, white cheese (200g)	0.320	[12]
Plastic/glass bottle	Oils, dressings, sauces	0.321	[100,106]
Glass jar	Pickled vegetables, marmalade	0.341	[107]
Plastic pot/glass jar	Herring in brine, honey, syrup, chocolate/nut paste	0.355	[101]
Plastic/paper/ aluminum wrap	Butter, butter blends, margarine (250g)	0.380	[12]
Glass bottle/bag-in-box (plastic + carton)	Wine	0.391	[96]
Plastic tub	Cream cheese (200 g)	0.400	[12]
Plastic tub	Crème fraiche (200 g)	0.410	[12]
Metal can	Canned vegetables, fruit and fish	0.451	[105]
Metal can	Milk powder	0.730	[12]
Glass bottle	Spirits	0.770	[96]

[1] Similar to information from Danish Brewers' Association https://bryggeriforeningen.dk/wp-content/uploads/2020/03/Faktaark-samlet.pdf

Table S3. The CF (kg CO_2-eq) from cooking 1 kg of raw food.

Food	CF per kg raw food cooked, kg CO_2-eq/kg food [1]
Meat	0.216
Fish and egg	0.108 [2]
Vegetables & fruit	0.109
Potatoes	0.138
Rice, quinoa, bulgur	0.243
Pasta	0.364
Flour, all	0.134 [3]
Coffee/tea (1 l water)	0.055
Legumes (dried beans and chickpeas) [4]	0.219
Legumes (lentils, split peas) [5]	0.121

[1] Unavoidable waste (bones, peel etc.) assumed not cooked.
[2] Cooking time is estimated to be half of that for meat.
[3] All flours are given cooking value correspoding to "baking a cake"
[4] Boiling time 45-60 minutes
[5] Boiling time 15-20 minutes

Table S4. Estimated CF (kg CO_2-eq/kg) related to storage in supermarket and home.

Type of storage in supermarket and home	GHG emissions, kg CO_2-eq/kg food [1)]	Type of storage used per food group
100% dry	0.033	Coffee, sugar, rice, pasta, legumes, canned products, sweets, spices, condiments
80% dry and 20% cold	0.037	Fresh vegetables and fruit, potatoes, soft drink, alcohol
100% cold	0.055	Dairy products, eggs
80% dry and 20% frozen	0.079	Bread, bakery
80% cold and 20% frozen	0.097	Fresh meat and fish
100% frozen	0.263	Dessert (mainly ice cream), fish, vegetables etc.

1) Sum of CF from storage in supermarket and home

Table S5. Total carbon footprint (CF) of the current diet among adults (18-64 year), per person per day and per 10 MJ, respectively*, calculated using three different CF data sets at household level.

	CF, AU-DTU data			CF, BCD excl. iLUC			CF, BCD incl. iLUC		
	**Average N=2492**	**Men N=1202**	**Women N=1290**	**Average N=2492**	**Men N=1202**	**Women N=1209**	**Average N=2492**	**Men N=1202**	**Women N=1290**
**CF kg CO_2-eq/pers/day**									
Mean	4.62[a]	5.29	4.00***	5.11[b]	6.04	4.26***	5.84[c]	6.91	4.83***
(SD)	(1.54)	(1.57)	(1.20)	(2.29)	(2.44)	(1.75)	(2.71)	(2.89)	(2.07)
Median	4.40	5.15	3.87	4.65	5.59	3.95	5.28	6.37	4.49
(P10;P90)	(2.95; 6.57)	(3.52; 7.21)	(2.67; 5.36)	(2.75; 8.10)	(3.42; 9.17)	(2.47; 6.28)	(3.06; 9.34)	(3.80; 10.60)	(2.76; 7.21)
**CF kg CO_2-eq/10 MJ**									
Mean	4.78[a]	4.78	4.78	5.29[b]	5.49	5.11***	6.04[c]	6.29	5.81***
(SD)	(0.96)	(0.91)	(1.01)	(1.98)	(1.97)	(1.97)	(2.38)	(2.37)	(2.37)
Median	4.62	4.65	4.61	4.89	5.08	4.72	5.56	5.80)	5.32
(P10;P90)	(3.72; 6.06)	(3.76; 5.93)	(3.69; 6.11)	(3.35; 7.69)	(3.46; 8.05)	(3.25; 7.31)	(3.71; 8.87)	(3.87; 9.38	(3.61; 8.47)

*) Average energy intake per day for the entire adult population 18-64 years is 9.81 MJ (3.12 MJ), and 11.24 MJ (3.24 MJ) and 8.49 MJ (2.32 MJ) for men and women, respectively (SD, standard deviation, in brackets). Significant difference between results of the total population in the same row is indicated with different letters a, b, c ($p<0.01$). Significant difference between gender is indicated: ***=$p<0.001$.

The current food intake: The Danish National Survey on Dietary habits and physical Activity (DANSDA) 2011-2013, adults 18-64 years. The AU-DTU data are compiled by researchers from Aarhus University and DTU (Technical University of Denmark) described in methodological section 2.3 of the present study. The BCD (the Big Climate Database): published by the Danish green think tank CONCITO. iLUC: indirect land use change.

References

1. Nguyen, T.L.T.; Hermansen, J.E.; Mogensen, L. *Environmental assessment of Danish Pork*; Aarhus University, Department of Agroecology: Tjele, Denmark, 2011; ISBN 978-87-91949-5-48.
2. Moberg, E.; Walker Andersson, M.; Säll, S.; Hansson, P.A.; Röös, E. Determining the climate impact of food for use in a climate tax—design of a consistent and transparent model. *Int. J. Life Cycle Assess.* **2019**, *24*, 1715–1728, doi:10.1007/s11367-019-01597-8.
3. Mogensen, L.; Hermansen, J.E.; Trolle, E. The Climate and Nutritional Impact of Beef in Different Dietary Patterns in Denmark. *Foods* **2020**, *9*, 1176, doi:10.3390/foods9091176.
4. Mogensen, L.; Nguyen, T.L.T.; Madsen, N.T.; Pontoppidan, O.; Preda, T.; Hermansen, J.E. Environmental impact of beef sourced from different production systems - focus on the slaughtering stage: input and output. *J. Clean. Prod.* **2016**, *133*, 284–293, doi:10.1016/j.jclepro.2016.05.105.
5. Clune, S.; Crossin, E.; Verghese, K. Systematic review of greenhouse gas emissions for different fresh food categories. *J. Clean. Prod.* **2017**, *140*, 766–783, doi:10.1016/j.jclepro.2016.04.082.
6. Nielsen, P.; Nielsen, A.; Weidema, B.; Dalgaard, R.; Halberg, N. LCA Food Database. Available online: www.lcafood.dk (accessed on Sep 1, 2021).
7. Jensen, J.K.; Arlbjørn, J.S. Product carbon footprint of rye bread. *J. Clean. Prod.* **2014**, *82*, 45–57, doi:10.1016/j.jclepro.2014.06.061.
8. Heusala, H.; Sinkko, T.; Mogensen, L.; Knudsen, M.T. Carbon footprint and land use of food products containing oat protein concentrate. *J. Clean. Prod.* **2020**, *276*, 122938, doi:10.1016/j.jclepro.2020.122938.
9. Kasmaprapruet, S.; Paengjuntuek, W.; Saikhwan, P.; Phungrassami, H. Life cycle assessment of milled rice production: case study in Thailand. *Eur. J. Sci. Res.* **2009**, *30*, 195–203.
10. Jeswani, H.K.; Burkinshaw, R.; Azapagic, A. Environmental sustainability issues in the food-energy-water nexus: Breakfast cereals and snacks. *Sustain. Prod. Consum.* **2015**, *2*, 17–28, doi:10.1016/j.spc.2015.08.001.
11. Tynelius, G. *Climate impact from Lantmännen Unibake´s Danish pastry Vanilla crown*; Lantmännen, Stockholm, Sweden, 2009;
12. Flysjö, A. Greenhouse gas emissions in milk and dairy product chains. Improving the carbon footprint of dairy products. PhD Thesis, Aarhus University, Tjele, Denmark, 2012.
13. Cerutti, A.K.; Contu, S.; Ardente, F.; Donno, D.; Beccaro, G.L. Carbon footprint in green public procurement: Policy evaluation from a case study in the food sector. *Food Policy* **2016**, *58*, 82–93, doi:10.1016/j.foodpol.2015.12.001.
14. Cederberg, C.; Wivstad, M.; Bergkvist, P.; Mattsson, B.; Ivarsson, K. *Hållbart växtskydd. Analys av olika strategier för att minska riskerna med kemiska växtskyddsmedel*; Sveriges Lantbruksuniversitet: Uppsala, Sweden, 2005;
15. González, A.D.; Frostell, B.; Carlsson-Kanyama, A. Protein efficiency per unit energy and per unit greenhouse gas emissions: Potential contribution of diet choices to climate change mitigation. *Food Policy* **2011**, *36*, 562–570, doi:10.1016/j.foodpol.2011.07.003.
16. Davis, J.; Wallman, M.; Sund, V.; Emanuelsson, A.; Cederberg, C.; Sonesson, U. *Emissions of Greenhouse Gases from Production of Horticultural Products Analysis of 17 products cultivated in Sweden*; The Swedish Institute for Food and Biotechnology (SIK) Gothenburg, Sweden, 2011; ISBN 9789172903012.
17. Canals, L.M.I.; Muñoz, I.; Hospido, A.; Plassmann, K.; McLaren, S. Life Cycle Assessment (LCA) of domestic vs. imported vegetables. Case studies on broccoli, salad crops and green beans. *United Kingdom, Cent. Environ. Strateg. Univ. Surrey*, Guildford, UK,**2008**, 46.
18. Stoessel, F.; Juraske, R.; Pfister, S.; Hellweg, S. Life cycle inventory and carbon and water foodprint of fruits and vegetables: Application to a swiss retailer. *Environ. Sci. Technol.* **2012**, *46*, 3253–3262, doi:10.1021/es2030577.
19. Sanyé-Mengual, E.; Oliver-Solà, J.; Antón, A.; Montero, J.I.; Rieradevall, J. *Environmental assessment of urban horticulture structures: Implementing Rooftop Greenhouses in Mediterranean cities*; In Proceedings of the 9th International Conference on Life

Cycle Assessment in the Agri-Food Sector, San Francisco, CA, USA, 8–10 October 2014; pp. 1169–1178.

20. Torrellas, M.; Antón, A.; López, J.C.; Baeza, E.J.; Parra, J.P.; Muñoz, P.; Montero, J.I. LCA of a tomato crop in a multi-Tunnel greenhouse in Almeria. *Int. J. Life Cycle Assess.* **2012**, *17*, 863–875, doi:10.1007/s11367-012-0409-8.
21. Torrellas, M.; Antón, A.; Ruijs, M.; García Victoria, N.; Stanghellini, C.; Montero, J.I. Environmental and economic assessment of protected crops in four European scenarios. *J. Clean. Prod.* **2012**, *28*, 45–55, doi:10.1016/j.jclepro.2011.11.012.
22. Halberg, N.; Dalgaard, R.; Rasmussen, M.D. *Miljøvurdering af konventionel og økologisk avl af grøntsager - Livscyklusvurdering af produktion i væksthuse og på friland: Tomater, agurker, løg, gulerødder*; Arbejdsraport fra Miljøstyrelsen nr 5. Ministry of the Environment, Copenhagen, Denmark, 2006;
23. Landquist, B.; Woodhouse, A. *Klimatavtryck av rotfrukter, grönsaker och kryddor Analys av tio produkter odlade i Sverige*; The Swedish Institute for Food and Biotechnology (SIK) Gothenburg, Sweden, 2015; ISBN 9789172903470.
24. Maraseni, T.N.; Cockfield, G.; Maroulis, J.; Chen, G. An assessment of greenhouse gas emissions from the Australian vegetables industry. *J. Environ. Sci. Health. B.* **2010**, *45*, 578–588, doi:10.1080/03601234.2010.493497.
25. Bell, E.M.; Stokes-Draut, J.R.; Horvath, A. Environmental evaluation of high-value agricultural produce with diverse water sources: Case study from Southern California. *Environ. Res. Lett.* **2018**, *13*, 25007, doi:10.1088/1748-9326/aaa49a.
26. Mogensen, L.; Knudsen, M.T.; Dorca-Preda, T.; Nielsen, N.I.; Kristense, I.S.; Kristensen, T. *Baeredygtighedsparametre for Konventionelle Fodermidler Til Kvaeg - Metode Og Tabelvaerdier*; DCA - Nationalt Center for Fødevarer og Jordbrug, Aarhus University, Tjele, Denmark, 2018;
27. Hospido, A.; Milà I Canals, L.; McLaren, S.; Truninger, M.; Edwards-Jones, G.; Clift, R. The role of seasonality in lettuce consumption: A case study of environmental and social aspects. *Int. J. Life Cycle Assess.* **2009**, *14*, 381–391, doi:10.1007/s11367-009-0091-7.
28. Tamburini, E.; Pedrini, P.; Marchetti, M.G.; Fano, E.A.; Castaldelli, G. Life cycle based evaluation of environmental and economic impacts of agricultural productions in the Mediterranean area. *Sustain.* **2015**, *7*, 2915–2935, doi:10.3390/su7032915.
29. Audsley, E.; Brander, M.; Chatterton, J.; Murphy-Bokern, D.; Webster, C.; Williams, A. *How low can we go? An assessment of greenhouse gas emissions from the UK food system and the scope for reduction by 2050*; W WF-UK , 2009;
30. Cellura, M.; Ardente, F.; Longo, S. From the LCA of food products to the environmental assessment of protected crops districts: A case-study in the south of Italy. *J. Environ. Manage.* **2012**, *93*, 194–208, doi:10.1016/j.jenvman.2011.08.019.
31. Landquist, B. *Jämförelse av klimatpåverkan för ekologiskt resp. IP-odlade gröna ärter*; The Swedish Institute for Food and Biotechnology (SIK) Gothenburg, Sweden, 2012;
32. Sonesson, U.; Cederberg, C.; Wivstad, M.; Florén, B. *Minskade risker med bekämpningsmedel och minskad miljöpåverkan, samtidigt? - En fallstudie på Findus konservärtsodling 1980-2005*; The Swedish Institute for Food and Biotechnology (SIK) Gothenburg, Sweden, 2007;
33. Potter, H.K.; Lundmark, L.; Röös, E. *Environmental impact of plant-based foods -Data collection for the development of a consumer guide for plant-based foods. Title of series: Report 112*; Swedish University of Agricultural Sciences, NL Faculty/ Department of Energy and Technology, Uppsala, Sweden, 2020; ISBN 978-91-576-9789-9 (elektronisk).
34. Meul, M.; Ginneberge, C.; Van Middelaar, C.E.; de Boer, I.J.M.; Fremaut, D.; Haesaert, G. Carbon footprint of five pig diets using three land use change accounting methods. *Livest. Sci.* **2012**, *149*, 215–223, doi:10.1016/j.livsci.2012.07.012.
35. Torres, C.M.; Antón, A.; Castellas, F. Moving toward scientific LCA for farmers. *Proc. 9th Int. Conf. Life Cycle Assess. Agri-Food Sect. (LCA Food 2014)* **2014**.
36. Blonk, H.; Kool, A.; Luske, B.; Ponsioen, T.; Scholten, J. Methodology for assessing carbon footprints of horticultural products horticultural products. Blonk Milieu Advies: CA Gouda, The Netherlands, **2010**.
37. Borzęcka, M.; Żyłowska, K.; Russo, G.; Pisanelli, A.; Freire, F. Life Cycle Assessment of olive cultivation in Italy: comparison of three management systems. *167th EAAE Semin. Eur. Agric. Transit. to Bioeconomy*, Puławy, Poland, 24–25 September, **2018**, 1–7.

38. De Gennaro, B.; Notarnicola, B.; Roselli, L.; Tassielli, G. Innovative olive-growing models: An environmental and economic assessment. *J. Clean. Prod.* **2012**, *28*, 70–80, doi:10.1016/j.jclepro.2011.11.004.
39. Aguilera, E.; Guzmán, G.; Alonso, A. Greenhouse gas emissions from conventional and organic cropping systems in Spain. II. Fruit tree orchards. *Agron. Sustain. Dev.* **2015**, *35*, 725–737, doi:10.1007/s13593-014-0265-y.
40. Romero-Gámez, M.; Suárez-Rey, E.M.; Antón, A.; Castilla, N.; Soriano, T. Environmental impact of screenhouse and open-field cultivation using a life cycle analysis: The case study of green bean production. *J. Clean. Prod.* **2012**, *28*, 63–69, doi:10.1016/j.jclepro.2011.07.006.
41. Jungbluth, N.; Keller, R.; König, A. ONE TWO WE—life cycle management in canteens together with suppliers, customers and guests. *Int. J. Life Cycle Assess.* **2016**, *21*, 646–653, doi:10.1007/s11367-015-0982-8.
42. Schäfer, F.; Blanke, M. Farming and marketing system affects carbon and water footprint - A case study using Hokaido pumpkin. *J. Clean. Prod.* **2012**, *28*, 113–119, doi:10.1016/j.jclepro.2011.08.019.
43. Robinson, B.; Winans, K.; Kendall, A.; Dlott, J.; Dlott, F. A life cycle assessment of Agaricus bisporus mushroom production in the USA. *Int. J. Life Cycle Assess.* **2019**, *24*, 456–467, doi:10.1007/s11367-018-1456-6.
44. Leiva, F.J.; Saenz-Díez, J.C.; Martínez, E.; Jiménez, E.; Blanco, J. Environmental impact of Agaricus bisporus cultivation process. *Eur. J. Agron.* **2015**, *71*, 141–148, doi:10.1016/j.eja.2015.09.013.
45. Gunady, M.G.A.; Biswas, W.; Solah, V.A.; James, A.P. Evaluating the global warming potential of the fresh produce supply chain for strawberries, romaine/cos lettuces (Lactuca sativa), and button mushrooms (Agaricus bisporus) in Western Australia using life cycle assessment (LCA). *J. Clean. Prod.* **2012**, *28*, 81–87, doi:10.1016/j.jclepro.2011.12.031.
46. Saunders, C.; Barber, A.; Taylor, G. *Food Miles-Comparative Energy/Emissions Performance of New Zealand's Agriculture Industry*; The Agribusiness and Economics Research Unit (AERU), Lincoln University, New Zealand, 2006;
47. Venkat, K. Comparison of Twelve Organic and Conventional Farming Systems: A Life Cycle Greenhouse Gas Emissions Perspective. *J. Sustain. Agric.* **2012**, *36*, 620–649, doi:10.1080/10440046.2012.672378.
48. Figueiredo, F.; Castanheira, É.G.; Feliciano, M.; Rodrigues, M.Â.; Peres, A.; Maia, F.; Ramos, A.; Carneiro, J.; Coroama, V.C.; Freire, F.; et al. Carbon footprint of apple and pear: orchards, storage and distribution. In Proceedings of the Energy for Sustainability 2013 Sustainable Cities: Designing for People and the Planet; 2013.
49. Yan, M.; Cheng, K.; Yue, Q.; Yan, Y.; Rees, R.M.; Pan, G. Farm and product carbon footprints of China's fruit production—life cycle inventory of representative orchards of five major fruits. *Environ. Sci. Pollut. Res.* **2016**, *23*, 4681–4691, doi:10.1007/s11356-015-5670-5.
50. Vinyes, E.; Gasol, C.M.; Asin, L.; Alegre, S.; Muñoz, P. Life Cycle Assessment of multiyear peach production. *J. Clean. Prod.* **2015**, *104*, 68–79, doi:10.1016/j.jclepro.2015.05.041.
51. Basset-Mens, C.; Vanniere, H.; Grasselly, D.; Heitz, H.; Braun, A.R.; Payen, S.; Koch, P. Environmental impacts of imported versus locally-grown fruits for the French market as part of the AGRIBALYSE® program.; In Proceedings of the 9th International Conference on Life Cycle Assessment in the Agri-Food Sector (LCA Food 2014), San Francisco, CA, USA 8-10 October, American Center for Life Cycle Assessment, 2014, pp 78-87.
52. Renz, B.; Pavlenko, N.; Acharya, A.; Jemison, C.; Lizas, D.; Kollar, T. Estimating energy and greenhouse gas emission savings through food waste source reduction. In Proceedings of the Proceedings of the 9th International Conference on Life Cycle Assessment in the Agri-Food Sector (LCA Food 2014); San Francisco, CA, USA, 8–10 October; 2014; pp. 8–10.
53. Pergola, M.; D'Amico, M.; Celano, G.; Palese, A.M.; Scuderi, A.; Di Vita, G.; Pappalardo, G.; Inglese, P. Sustainability evaluation of Sicily's lemon and orange production: Anenergy, economic and environmental analysis. *J. Environ. Manage.* **2013**, *128*, 674–682, doi:10.1016/j.jenvman.2013.06.007.
54. Knudsen, M.T.; Fonseca de Almeida, G.; Langer, V.; Santiago de Abreu, L.; Halberg, N. Environmental assessment of organic juice imported to Denmark: A case study on oranges (Citrus sinensis) from Brazil. *Org. Agric.* **2011**, *1*, 167–185, doi:10.1007/s13165-011-0014-3.

55. Doublet, G.; Jungbluth, N.; Schori, M.; Salome, S. Life cycle assessment of orange juice. *Harmon. Environ. Sustain. Eur. food Drink Chain, Project no 288974, EC Funded, Deliverable D2.1 ESU-Services Ltd. Zürich, Switzerland,* **2013**, 1–38.
56. Beccali, M.; Cellura, M.; Iudicello, M.; Mistretta, M. Resource consumption and environmental impacts of the agrofood sector: Life cycle assessment of italian citrus-based products. *Environ. Manage.* **2009**, *43*, 707–724, doi:10.1007/s00267-008-9251-y.
57. Ribal, J.; Ramírez-Sanz, C.; Estruch, V.; Clemente, G.; Sanjuán, N. Organic versus conventional citrus. Impact assessment and variability analysis in the Comunitat Valenciana (Spain). *Int. J. Life Cycle Assess.* **2017**, *22*, 571–586, doi:10.1007/s11367-016-1048-2.
58. Cichelli, A.; Pattara, C.; Petrella, A. Sustainability in Mountain Viticulture. The Case of the Valle Peligna. *Agric. Agric. Sci. Procedia* **2016**, *8*, 65–72, doi:10.1016/j.aaspro.2016.02.009.
59. Bartocci, P.; Fantozzi, P.; Fantozzi, F. Environmental impact of Sagrantino and Grechetto grapes cultivation for wine and vinegar production in central Italy. *J. Clean. Prod.* **2017**, *140*, 569–580, doi:10.1016/j.jclepro.2016.04.090.
60. Falcone, G.; De Luca, A.I.; Stillitano, T.; Strano, A.; Romeo, G.; Gulisano, G. Assessment of environmental and economic impacts of vine-growing combining life cycle assessment, life cycle costing and multicriterial analysis. *Sustain.* **2016**, *8*, 793, doi:10.3390/su8080793.
61. Moberg, E.; Potter, H.K.; Wood, A.; Hansson, P.-A.A.; Röös, E. Benchmarking the Swedish diet relative to global and national environmental targets-Identification of indicator limitations and data gaps. *Sustain.* **2020**, *12*, 1407, doi:10.3390/su12041407.
62. De Ramos, R.M.Q.; Taboada, E.B. Cradle-to-gate life cycle assessment of fresh and processed pineapple in the Philippines. *Nat. Environ. Pollut. Technol.* **2018**, *17*, 783–790.
63. Ingwersen, W.W. Life cycle assessment of fresh pineapple from Costa Rica. *J. Clean. Prod.* **2012**, *35*, 152–163.
64. Usubharatana, P.; Phungrassami, H. Evaluation of Opportunities to Reduce the Carbon Footprint of Fresh and Canned Pineapple Processing in Central Thailand. *Polish J. Environ. Stud.* **2017**, *26, 1725-1735.*
65. Müller, K.; Holmes, A.; Deurer, M.; Clothier, B.E. Eco-efficiency as a sustainability measure for kiwifruit production in New Zealand. *J. Clean. Prod.* **2015**, *106*, 333–342, doi:10.1016/j.jclepro.2014.07.049.
66. McLaren, S.J.; Hume, A.; Nalanie Mitraratne *Carbon management for the primary agricultural sector in new Zealand: case studies for the pipfruit and kiwifruit industries*; Proceeding of the VII International Conference on Food LCA, 2010; Vol. 1, 293-298.
67. Mithraratne, N.; Barber, A.; McLaren, S.J. *Carbon Footprinting for the Kiwifruit Supply Chain – Report on Methodology and Scoping Study Final Report*; New Zealand LifeCycle Management Centre, Massey University, New Zealand, 2010;
68. Pergola, M.; Persiani, A.; Pastore, V.; Palese, A.M.; Arous, A.; Celano, G. A comprehensive Life Cycle Assessment (LCA) of three apricot orchard systems located in Metapontino area (Southern Italy). *J. Clean. Prod.* **2017**, *142*, 4059–4071, doi:10.1016/j.jclepro.2016.10.030.
69. Tassielli, G.; Notarnicola, B.; Renzulli, P.A.; Arcese, G. Environmental life cycle assessment of fresh and processed sweet cherries in southern Italy. *J. Clean. Prod.* **2018**, *171*, 184–197, doi:10.1016/j.jclepro.2017.09.227.
70. Bravo, G.; López, D.; Vásquez, M.; Iriarte, A. Carbon Footprint Assessment of Sweet Cherry Production: Hotspots and Improvement Options. *Pol. J. Environ. Stud* **2017**, *26*, 559–566, doi:10.15244/pjoes/65361.
71. Webb, J.; Williams, A.G.; Hope, E.; Evans, D.; Moorhouse, E. Do foods imported into the UK have a greater environmental impact than the same foods produced within the UK? *Int. J. Life Cycle Assess.* **2013**, *18*, 1325–1343, doi:10.1007/s11367-013-0576-2.
72. Williams, A.; Pell, E.; Webb, J.; Moorhouse, E.; Audsley, E. Strawberry and tomato production for the UK compared between the UK and Spain. In Proceedings of the 6th International Conf. on LCA in the Agri-Food Sector, Zürich; 2008; pp. 254–414.
73. Girgenti, V.; Peano, C.; Bounous, M.; Baudino, C. A life cycle assessment of non-renewable energy use and greenhouse gas emissions associated with blueberry and raspberry production in northern Italy. *Sci. Total Environ.* **2013**, *458–460*, 414–418, doi:10.1016/j.scitotenv.2013.04.060.
74. Peano, C.; Baudino, C.; Tecco, N.; Girgenti, V. Green marketing tools for fruit growers associated groups: Application of the

Life Cycle Assessment (LCA) for strawberries and berry fruits ecobranding in northern Italy. *J. Clean. Prod.* **2015**, *104*, 59–67, doi:10.1016/j.jclepro.2015.04.087.

75. Cordes, H.; Iriarte, A.; Villalobos, P. Evaluating the carbon footprint of Chilean organic blueberry production., doi:10.1007/s11367-016-1034-8.
76. Abeliotis, K.; Detsis, V.; Pappia, C. Life cycle assessment of bean production in the Prespa National Park, Greece. *J. Clean. Prod.* **2013**, *41*, 89–96, doi:10.1016/j.jclepro.2012.09.032.
77. Mejia, M.; Fresán, U.; Harwatt, H.; Oda, K.; Uriegas-Mejia, G.; Sabaté, J. Life Cycle Assessment of the Production of a Large Variety of Meat Analogs by Three Diverse Factories. *J. Hunger Environ. Nutr.* **2020**, *15*, 699–711, doi:10.1080/19320248.2019.1595251.
78. Blonk, H.; Kool, A.; Luske, B. Milieueffecten van Nederlandse consumptie van eiwitrijke producten Gevolgen van vervanging van dierlijke eiwitten anno 2008. Blonk Milieu Advies: CA Gouda, The Netherlands, **2008**.
79. Blonk, H.; Kool, A.; Luske, B.; Waart, S. De Environmental effects of protein-rich food products in the Netherlands Consequences of animal protein substitutes. Blonk Milieu Advies: CA Gouda, The Netherlands, **2008**, 1–19.
80. Nemecek, T.; Weiler, K.; Plassmann, K.; Schnetzer, J.; Gaillard, G.; Jefferies, D.; García-Suárez, T.; King, H.; Milà I Canals, L. Estimation of the variability in global warming potential of worldwide crop production using a modular extrapolation approach. *J. Clean. Prod.* **2012**, *31*, 106–117, doi:10.1016/j.jclepro.2012.03.005.
81. Volpe, R.; Messineo, S.; Volpe, M.; Messineo, A. Carbon footprint of tree nuts based consumer products. *Sustain.* **2015**, *7*, 14917–14934, doi:10.3390/su71114917.
82. Bartzas, G.; Vamvuka, D.; Komnitsas, K. Comparative life cycle assessment of pistachio, almond and apple production. *Inf. Process. Agric.* **2017**, *4*, 188–198, doi:10.1016/j.inpa.2017.04.001.
83. Buchspies, B.; Tölle, S.; Jungbluthy, N. Life Cycle Assessment of High-Sea Fish and Salmon Aquaculture. *Report* **2011**, 20.
84. Parker, R.W.R.; Vázquez-Rowe, I.; Tyedmers, P.H. Fuel performance and carbon footprint of the global purse seine tuna fleet. *J. Clean. Prod.* **2015**, *103*, 517–524, doi:10.1016/j.jclepro.2014.05.017.
85. Ziegler, F.; Winther, U.; Hognes, E.S.; Emanuelsson, A.; Sund, V.; Ellingsen, H. The Carbon Footprint of Norwegian Seafood Products on the Global Seafood Market. *J. Ind. Ecol.* **2013**, *17*, 103–116, doi:10.1111/j.1530-9290.2012.00485.x.
86. Winther, U.; Hognes, E.S.; Jafarzadeh, S.; Ziegler, F. *Greenhouse gas emissions of Norwegian seafood products in 2017*; SINTEF Ocean AS, Trondheim, Norway, 2020; ISBN 9788214062465.
87. Ziegler, F.; Valentinsson, D. Environmental life cycle assessment of Norway lobster (Nephrops norvegicus) caught along the Swedish west coast by creels and conventional trawls - LCA methodology with case study. *Int. J. Life Cycle Assess.* **2008**, *13*, 487–497, doi:10.1007/s11367-008-0024-x.
88. Wallén, A.; Brandt, N.; Wennersten, R. Does the Swedish consumer's choice of food influence greenhouse gas emissions? *Environ. Sci. Policy* **2004**, *7*, 525–535, doi:10.1016/J.ENVSCI.2004.08.004.
89. Röös, E. *Mat-klimat-listan Version 1.1*; Swedish University of Agricutural Sciences, Uppsala, Sweden, 2014;
90. Heller, M.C.; Willits-Smith, A.; Meyer, R.; Keoleian, G.A.; Rose, D. Greenhouse gas emissions and energy use associated with production of individual self-selected US diets. *Environ. Res. Lett.* **2018**, *13*, 044004, doi:10.1088/1748-9326/aab0ac.
91. World Resources Institute Cool Food Pledge Calculator - Version April 8, 2020 Available online: https://www.wri.org/research/tracking-progress-toward-cool-food-pledge (accessed on Oct 15, 2021).
92. Poore, J.; Nemecek, T. Reducing food's environmental impacts through producers and consumers. *Science (80-.).* **2018**, *360*, 987–992, doi:10.1126/science.aaq0216.
93. Kendall, A.; Yuan, J.; Brodt, S.B. Carbon footprint and air emissions inventories for US honey production: Case studies. *Int. J. Life Cycle Assess.* **2013**, *18*, 392–400, doi:10.1007/s11367-012-0487-7.
94. Nilsson, K.; Sund, V.; Floren, B. *The environmental impact of the consumption of sweets, crisps and soft drinks*; TemaNord 2011:509 Nordic Council of Ministers, Copenhagen, Denmark,, 2011; ISBN 9789289321976.

95. Bryggeriforeningen Faktaark. Klimaaftryk Available online: https://bryggeriforeningen.dk/wp-content/uploads/2020/03/Faktaark-samlet.pdf. (accessed on 20 February 2021).
96. Hallström, E.; Håkansson, N.; Åkesson, A.; Wolk, A.; Sonesson, U. Climate impact of alcohol consumption in Sweden. *J. Clean. Prod.* **2018**, *201*, 287–294, doi:10.1016/j.jclepro.2018.07.295.
97. Frankowska, A.; Jeswani, H.K.; Azapagic, A. Environmental impacts of vegetables consumption in the UK. *Sci. Total Environ.* **2019**, *682*, 80–105, doi:10.1016/j.scitotenv.2019.04.424.
98. Hartikainen, H.; Pulkkinen, H. *Summary of the chosen methodologies and practices to produce GHGE-estimates for an average European diet*; Natural Resources Institute Finland: Helsinki, Finland, 2016; ISBN 9789523263130.
99. Public Health England *McCance and Widdowson's composition of foods integrated dataset*; 2021;
100. Wallman, M.; Nilsson, K. Klimatpåverkan och energianvändning från livsmedelsförpackningar. Livsmedelsverket, Uppsala, Sweden, 2011.
101. *Comparative Life Cycle Assessment of sterilised food packagning systems on the European market*; Institut für Energi- und Unweltforschung (IFEU), Heidelberg, Germany, 2013;
102. Heller, M.C.; Selke, S.E.M.; Keoleian, G.A. Mapping the Influence of Food Waste in Food Packaging Environmental Performance Assessments. *J. Ind. Ecol.* **2019**, *23*, 480–495, doi:10.1111/jiec.12743.
103. Maga, D.; Hiebel, M.; Aryan, V. A comparative life cycle assessment of meat trays made of various packaging materials. *Sustain.* **2019**, *11*, doi:10.3390/su11195324.
104. WRAP *Methodology for assessing the climate change impacts of packaging optimisation under the Courtauld Commitment 3*; The Waste and Resources Action Programme (WRAP), Banbury, UK, 2014;
105. Markwardt, S.; Wellenreuther, F. Key findings of LCA study on Tetra Recart 2017. Institute for Energy and Environmental Research, Heidelberg, Germany, 2017
106. Del Borghi, A.; Gallo, M.; Strazza, C.; Del Borghi, M. An evaluation of environmental sustainability in the food industry through Life Cycle Assessment: The case study of tomato products supply chain. *J. Clean. Prod.* **2014**, *78*, 121–130, doi:10.1016/j.jclepro.2014.04.083.
107. Manfredi, M.; Vignali, G. Life cycle assessment of a packaged tomato puree: A comparison of environmental impacts produced by different life cycle phases. *J. Clean. Prod.* **2014**, *73*, 275–284, doi:10.1016/j.jclepro.2013.10.010.
